# Inference of ventricular activation properties from non-invasive electrocardiography

**DOI:** 10.1016/j.media.2021.102143

**Published:** 2021-10

**Authors:** Julia Camps, Brodie Lawson, Christopher Drovandi, Ana Minchole, Zhinuo Jenny Wang, Vicente Grau, Kevin Burrage, Blanca Rodriguez

**Affiliations:** a Department of Computer Science, University of Oxford, Oxford, United Kingdom; b Australian Research Council Centre of Excellence for Mathematical and Statistical Frontiers (ACEMS), Queensland University of Technology (QUT), Brisbane, Australia; c QUT Centre for Data Science (CDS), Queensland University of Technology, Brisbane, Australia; d Institute of Biomedical Engineering (IBME), University of Oxford, Oxford, United Kingdom

**Keywords:** Electrocardiographic imaging, Bayesian inference, Digital twin, Electrocardiogram

## Abstract

The realisation of precision cardiology requires novel techniques for the non-invasive characterisation of individual patients’ cardiac function to inform therapeutic and diagnostic decision-making. Both electrocardiography and imaging are used for the clinical diagnosis of cardiac disease. The integration of multi-modal datasets through advanced computational methods could enable the development of the cardiac ‘digital twin’, a comprehensive virtual tool that mechanistically reveals a patient's heart condition from clinical data and simulates treatment outcomes. The adoption of cardiac digital twins requires the non-invasive efficient personalisation of the electrophysiological properties in cardiac models. This study develops new computational techniques to estimate key ventricular activation properties for individual subjects by exploiting the synergy between non-invasive electrocardiography, cardiac magnetic resonance (CMR) imaging and modelling and simulation. More precisely, we present an efficient sequential Monte Carlo approximate Bayesian computation-based inference method, integrated with Eikonal simulations and torso-biventricular models constructed based on clinical CMR imaging. The method also includes a novel strategy to treat combined continuous (conduction speeds) and discrete (earliest activation sites) parameter spaces and an efficient dynamic time warping-based ECG comparison algorithm. We demonstrate results from our inference method on a cohort of twenty virtual subjects with cardiac ventricular myocardial-mass volumes ranging from 74 cm^3^ to 171 cm^3^ and considering low versus high resolution for the endocardial discretisation (which determines possible locations of the earliest activation sites). Results show that our method can successfully infer the ventricular activation properties in sinus rhythm from non-invasive epicardial activation time maps and ECG recordings, achieving higher accuracy for the endocardial speed and sheet (transmural) speed than for the fibre or sheet-normal directed speeds.

## Highlights

• Estimation of the ventricular speeds and earliest activation sites from ECG and CMR.
• Evaluation with twenty virtual subjects shows the effect of anatomical variability.
• Bayesian-inspired simultaneous estimation of continuous and discrete parameters.
• Efficient dynamic time warping-based comparison of electrocardiograms (ECG).
• Changing fibre and sheet-normal speed does not affect healthy activation sequence.

## Graphical abstract

**Figure:**
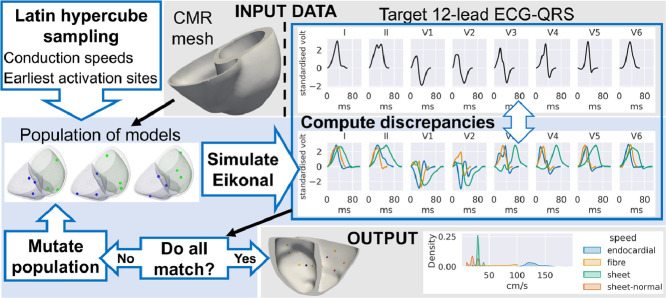

## 1 Introduction

Cardiovascular diseases are the most common non-communicable disease globally, accounting for 17.8 million deaths in 2017, according to the World Health Organisation (Kaptoge et al., 2019). Cardiac disease increases the risk of sudden and premature death through alterations in cardiac electrophysiology and tissue structure, which promote lethal arrhythmias and mechanical dysfunction.

Imaging and electrocardiographic datasets are commonly used for the diagnosis of cardiac disease. The electrocardiogram (ECG) is the most widely used clinical modality for cardiac disease diagnosis. Its interpretation is, however, confounded by anatomical and functional variability in the human population (Mincholé et al., 2019; Nguyên et al., 2015). Non-invasive imaging, through ultrasound, computerised tomography or cardiac magnetic resonance (CMR), is also used clinically to provide further information on cardiac anatomy, structure and mechanical function (Dall'Armellina et al., 2010; Tornvall et al., 2015; Dastidar et al., 2015; Biesbroek et al., 2018, 2019; Ferreira, 2019; Hausvater et al., 2019). Novel techniques are needed to fully exploit the synergy obtained by combining ECG and non-invasive clinical modalities such as CMR.

Recent studies have shown the power of patient-specific image-based modelling and simulation for therapy guidance, arrhythmic biomarkers interpretation and patient's phenotypic variability interpretation (Potse et al., 2014; Zettinig et al., 2014; Gillette et al., 2017; Kahlmann et al., 2017; Lyon et al., 2018; Bukhari et al., 2019; Niederer et al., 2019; Boyle et al., 2019; Martinez-Navarro et al., 2021). This technology has paved the way towards realising the ‘digital twin’ vision (Corral-Acero et al., 2020), referring to a comprehensive virtual tool that coherently integrates a patient's clinical data with mechanistic physiological knowledge and that can inform therapeutic and diagnostic decision-making through simulations. The generation of cardiac digital twin requires developing novel methodologies to analyse and estimate patient-specific properties from clinical test data, such as electrocardiography and CMR.

This study investigates new computational techniques for the efficient quantification of subject-specific ventricular activation properties using CMR-based modelling and simulation and non-invasive electrocardiographic data. We present an inference method combined with fast Eikonal-based simulations and CMR-based torso-biventricular anatomical models to determine the accuracy in the estimation of activation properties (such as endocardial and myocardial conduction speeds and the location of the Purkinje endocardial root nodes) from the QRS complex of the 12-lead ECG or activation time maps (as obtained through electrocardiographic imaging). We conduct the simultaneous inference of endocardial and myocardial conduction speeds and the root nodes (i.e. earliest activation sites), as these properties determine the activation sequence in the ventricles (Cardone-Noott et al., 2016). To address the challenges associated with inferring root node locations and speeds simultaneously, we implement a novel inference method based on the sequential Monte Carlo approximate Bayesian computation algorithm (SMC-ABC) (Appendix A.5) (Drovandi and Pettitt, 2011; Sisson et al., 2007). From our in-silico evaluations, we quantified the accuracy of recovering these activation properties from synthetic epicardial activation maps and 12-lead QRS complexes in a cohort of twenty virtual subjects, namely, twenty torso-biventricular models from four different anatomies and five electrophysiological configurations. Our analysis will aid future works addressing this previously unexplored, subject-specific calibration problem in both ventricles simultaneously, considering the physiological variability in the human population.

## 2 Materials and methods

### 2.1 Overview

**Fig. 1:**
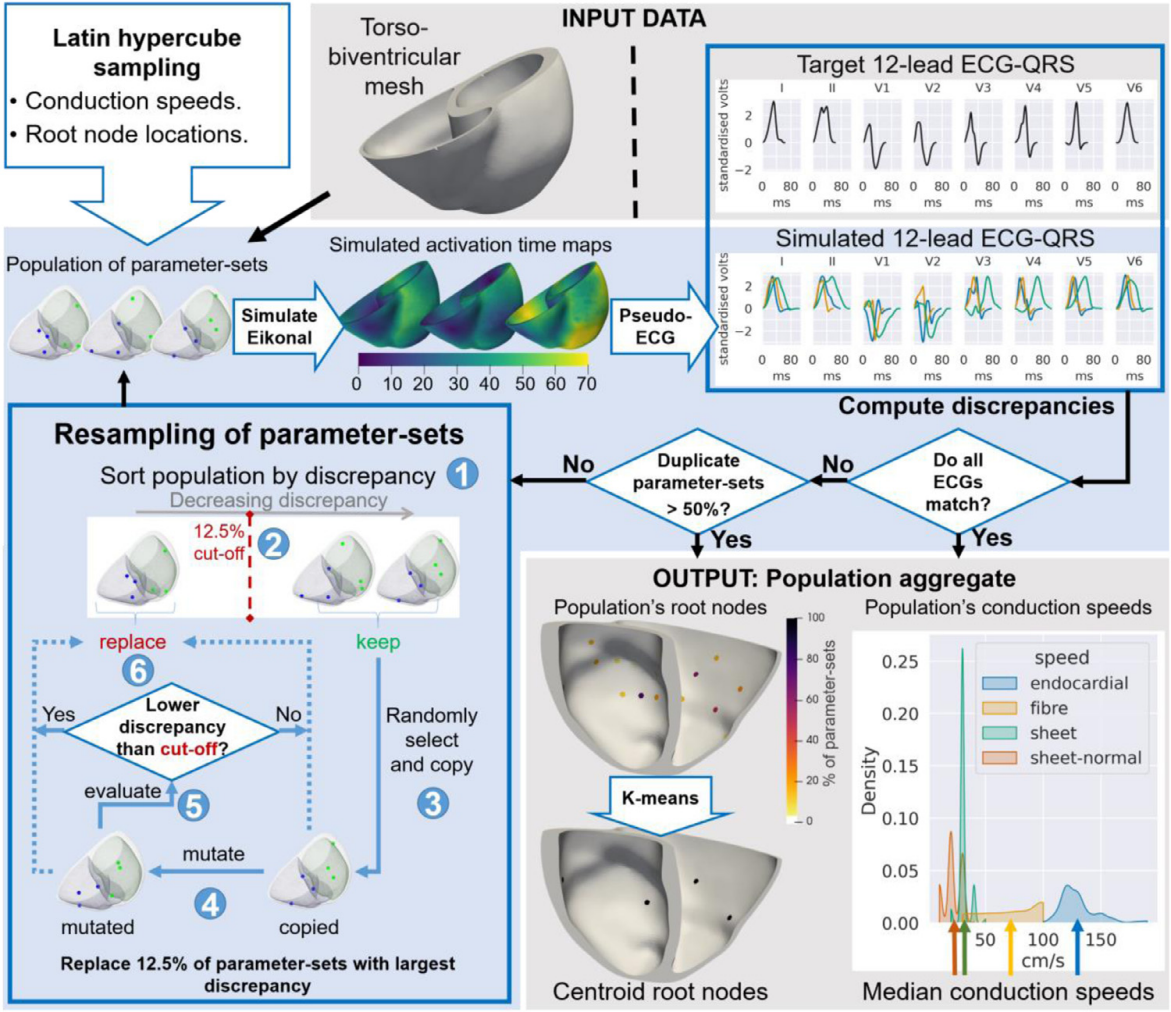
Proposed SMC-ABC based inference method. Diagram of our inference pipeline, and its subprocesses, to recover the ventricular activation properties from CMR and electrocardiographic data. The process starts with the Latin hypercube sampling (top-left), generating a population of 512 models with different parameter values but the same propagation model equations and a subject-specific CMR-based torso-biventricular mesh. From there, the iterative process (centre and bottom-left – blue shaded area) commences in the direction of the arrows until any of the two stopping criteria (centre-right rhomboidal boxes) are fulfilled. From top-left to the right, the diagram highlights the following subprocesses: simulation of activation maps using the parameter-sets in the Eikonal model, generation of the 12-lead ECGs through the pseudo-ECG algorithm, computation of the discrepancy between each prediction and the target 12-lead ECG-QRS, stopping criteria, and, if not terminating, modification of the current population of parameter-sets (bottom-left blue box), which will replace each parameter-set with the ‘high’ (top 12.5%) discrepancy by either a copy of a parameter-set with low discrepancy (dotted line – right) or a mutated version of this copy (dotted line – left). The input and output data (both shaded in grey) are depicted at the top-right and bottom-right parts of the figure, respectively.

Fig. 1 presents a diagram of our inference method, including input, output, and the iterative process to infer the human ventricular activation properties from electrocardiographic recordings using CMR-based modelling and simulation. The input data (top-right – grey shaded area) include ‘given data’ (torso-biventricular mesh and fibre orientations) as well as the ‘target data’ (e.g. 12-lead ECG recordings). The iterative process (centre and bottom-left – blue shaded area) aims to find a population of models (with different parameter-sets but the same equations and subject-specific anatomical model) that yield simulations in agreement with the ‘target data’ (bottom-right – grey shaded area). Each model in this population implements one set of parameter values (hereafter referred to as parameter-set), namely, a value for the locations of the root nodes (earliest activation sites), the endocardial layer's isotropic speed, and the fibre, sheet (transmural), and sheet-normal directed orthogonal conduction speeds.

The process depicted in Fig. 1 starts with the Latin hypercube sampling (Iman and Conover, 1980) (top-left), generating a population of 512 parameter-sets for conduction speeds and root nodes with a uniform prior. These parameter-sets are combined with the subject's CMR-based biventricular mesh and the Eikonal formulation to produce an initial population of 512 cardiac electrophysiological models. The iterative process followed for the inference (centre and bottom-left – blue shaded area) follows five steps (from the top-left on the directions of arrows in Fig. 1):

1 simulate the activation time maps from the new population of models;
2 calculate the predicted ECGs (unless when working directly on activation maps);
3 compute the discrepancies (distance) between each prediction and the target data (e.g. ECG);
4 evaluate the stopping criteria;
5 replace the parameter-sets corresponding to the top 12.5% highest discrepancies.

In Step-1, the Eikonal model (Appendix A.2) simulates an activation time map from each parameter-set in the population using the subject's torso-biventricular mesh. Next, Step-2 (not needed when inferring from activation maps) computes the ECG-QRS from each activation time map using the pseudo-ECG algorithm (Appendix A.4) (Gima and Rudy, 2002). Then, Step-3 computes the discrepancy (distance metric) between the predicted and target data (activation maps or ECG) (Section 2.4). Step-4 checks if any of the following stopping criteria (Section 2.6) are fulfilled:

1 all predicted data ‘match’ the target (using a threshold);
2 more than 50% of the parameter-sets in the population are duplicates.

If the population does not fulfil any criteria, the iterative process continues. The following six substeps compose Step-5 (Section 2.6) (see numbers on Fig. 1– bottom-left):

1 sort the parameter-sets in the population according to the discrepancy values (decreasing order);
2 define a discrepancy cut-off to replace the parameter-sets with the top 12.5% highest discrepancies in the population;
3 for each parameter-set to be replaced (within the 12.5%), randomly select one to be kept and create two new copies from it;
4 ‘mutate’ only one of each pair of copies using Markov Chain Monte Carlo (MCMC) (Gilks, 2005);
5 simulate the Eikonal model for the mutated parameter-sets and compute their discrepancies;
6 finally, replace each parameter-set in the top 12.5% by the mutated alternative (dotted line on the left), unless its discrepancy would also have been rejected by the cut-off, in which case, use the unmodified copy for the replacement (dotted line on the right).

After Step-5, the iterative process restarts from Step-1 with simulations using the modified population of parameter-sets, and the inference process carries on until any stopping criterion in Step-4 is met.

The output area in Fig. 1 (bottom right – grey shaded area) also illustrates the post-processing of the parameter-sets in the resulting population. We combined all parameter values in the population to calculate a single solution parameter-set for each inference. The strategy to combine our parameter-sets (Section 2.7) takes the median value for the conduction speeds and the k-means centroid for the root nodes throughout the population.

### 2.2 Virtual subjects for the ‘target data’

The ‘target data’ were produced synthetically in order to know the ground truth for the evaluation of the inference algorithm. The ground truth is not available through clinical datasets. They included epicardial activation maps and 12-lead ECGs generated through simulating CMR-based torso-biventricular models for twenty virtual subjects. These virtual subjects were obtained as described in Mincholé et al. (2019) through combinations of four torso-biventricular meshes constructed from the CMRs of four subjects and five conduction speed scenarios (Appendix A.3), as outlined in Table 1.

**Table 1:** The five conduction-speed configurations considered for this study. These speed values were selected to represent variability in the healthy human population (Appendix A.3), as in Mincholé et al. (2019).

Conduction configuration	Endocardial speed	Fibre speed	Sheet speed	Sheet-normal speed
Normal speeds	150 cm/s	50 cm/s	32 cm/s	29 cm/s
Slow endocardial speed	120 cm/s	50 cm/s	32 cm/s	29 cm/s
Fast endocardial speed	179 cm/s	50 cm/s	32 cm/s	29 cm/s
Fast endocardial and myocardial speeds	179 cm/s	88 cm/s	49 cm/s	45 cm/s
Slow endocardial and fast myocardial speeds	120 cm/s	88 cm/s	49 cm/s	45 cm/s

More precisely, we considered four torso-biventricular CMR-based meshes with a variable torso cavity and biventricular myocardial-mass volumes to explore the effects of anatomical variability. These four meshes had the following torso cavity volumes: 23,000 cm^3^, 27,000 cm^3^, 54,000 cm^3^, and 44,000 cm^3^ for Mesh-1, Mesh-2, Mesh-3, and Mesh-4, respectively. The biventricular myocardial-mass volumes for these meshes were 74 cm^3^, 76 cm^3^, 107 cm^3^, and 171 cm^3^.

### 2.3 Simulation protocols

While our inference method always simulated the Eikonal model, we considered two source models for the ‘target data’ to guide the inference process and provide grounds for its evaluation. Firstly, we considered noise-contaminated Eikonal simulations as ‘target data’, similarly to Grandits et al. (2020); and secondly, we considered the same cohort of virtual subjects simulated through the bidomain model, as in Mincholé et al. (2019).

The Eikonal model was simulated on CMR-based anatomical biventricular meshes and solved using Dijkstra's algorithm (Dijkstra, 1959) (Appendix A.2). We selected this algorithm due to its faster computational time yet equivalent simulated activation sequences to the fast marching method (Wallman et al., 2012). All models implemented rule-based fibre orientations (Streeter et al., 1969) with fibre angles transmurally changing from 60 to −60° from endocardium to epicardium, respectively. The electrical conduction speeds were defined as orthotropic in the myocardial tissue and isotropic in the endocardia (fast endocardial layer) to emulate the Purkinje network's effect. These modelling protocols were based on findings from Cardone-Noott et al. (2016) and Mincholé et al. (2019) on generating realistic healthy human 12-lead ECG recordings with torso-biventricular geometries.

The 12-lead ECG ‘target data’ were computed from the simulated activation maps using the pseudo-ECG algorithm (Appendix A.4) (Gima and Rudy, 2002) with electrode locations informed by torso geometry and orientation (Appendix A.3), as in Cardone-Noott et al. (2016) and Mincholé et al. (2019). The pseudo-ECG method provides a fast and simple evaluation of the normalised ECG without major loss of morphological information compared with bidomain simulations (Fig. A.1 in Appendix A.4) (Mincholé et al., 2019). Finally, we contaminated all ‘target data’ (epicardial activation maps and 12-lead ECGs) with white Gaussian noise to reach 20 dB of signal-to-noise ratio as in Almeida et al. (2004) and Potyagaylo et al. (2014).

Furthermore, a second set of ‘target data’ were obtained from bidomain (Appendix A.1) simulations without noise-contamination for comparison. The bidomain simulations were conducted using the Chaste software (Pitt-Francis et al., 2009) and the modified version of the O'Hara-Rudy action potential model (O'Hara et al., 2011) proposed by Dutta et al. (2017) (Appendix A.3). The 12-lead ECG ‘target data’ were computed from these bidomain-generated activation maps using the pseudo-ECG algorithm.

### 2.4 Discrepancy calculation

Measuring differences between epicardial activation maps to capture differences in the source activation properties is simple due to the sheared spatial representation of the epicardial activation map data (epicardium) and the root node locations (endocardium). Consequently, we defined the discrepancy between ‘predicted’ and ‘target’ epicardial activation maps as the root mean square error between the two activation maps. On the other hand, capturing differences in activation properties from differences between ECG recordings is challenging. Therefore, we propose a novel extension of the dynamic time warping (DTW) (Velichko and Zagoruyko, 1970) algorithm as the discrepancy metric for ECG data.

DTW is a speed-invariant dynamic-programming algorithm for measuring differences between sequences. Hence, DTW can compare signals of different lengths, such as ECGs (Potyagaylo et al., 2019; Ramírez et al., 2017), by stretching and shrinking them in the time axis. Classic DTW allows ECGs to be compared using non-physiological warping (Luzianin and Krause, 2016). Sakoe and Chiba (1978) proposed window-size and warping-slope constraints for DTW that restrict the maximum cumulative and per-step amount of warping along one ‘direction’ (either shrinking or stretching), respectively. Moreover, Itakura (1975) presented a similar window-size constraint but shaped like a parallelogram for speech recognition. This parallelogram constraint implied that the signals could warp less on the start, and it is usually complemented with a common-start and common-end constraints when both signals represent the same phenomena, giving rise to the parallelogram shape.

We assume that in healthy ventricles, the root node locations are primarily responsible for the QRS's morphology and that the conduction speeds mostly define the QRS's width, as demonstrated by Cardone-Noot et al. (2016). This assumption allows us to separate our optimisation process into two distinct responsibilities: 1) recovering the root nodes from the morphology of the 12-lead ECG-QRS while preserving all speed values; and, once all parameter-sets produce acceptable morphologies, 2) iteratively narrow down the population to the parameter-sets with the conduction speeds that generate the most similar QRS width to the target recording.

We present an extension of DTW that prevents non-physiological ECG transformations. Our DTW discrepancy implements the warping-slope constraint, a modified version of the parallelogram constraint, and a common-end warping constraint to avoid non-physiological ECG transformations. The warping-slope constraint prevents more than two warping steps from occurring consecutively (i.e. 1 ms can warp to up to 3 ms, and vice versa). The parallelogram constraint limits the amount of warping at the signals’ start and favours ‘diagonal’ warping such that one signal is transformed as the stretched or shrank version of the other. In other words, it rewards signals with physiologically similar morphology, namely, penalising non-monotonic warping, especially when occurring discontinuously or displaying ‘unwarping’ (i.e. stretching and shrinking different parts of the same signal within the same comparison). These warping penalties within the parallelogram are more prominent at the start of the signals and decrease linearly, allowing for more flexible warping when comparing the end of the QRSs since differences in the activation sequence can have a cumulative impact on the QRS. The common-start and common-end constraints imply that both signals represent the same phenomena and should be entirely encoded into each other. Overall, our DTW method favours signals with similar morphologies, even if their source conduction speeds were scaled to fast-track the root nodes’ identification. Therefore, we implement an additional penalty based on the difference in QRS-width to identify the source model's correct speeds, which, combined with the DTW extension described, composes our DTW-based discrepancy.

### 2.5 Parameter space exploration

Our approach aims to infer the root node locations and the four conduction speeds (i.e. endocardial, fibre, sheet, and sheet-normal speeds) that enable reproducing a subject's ECG recording (or epicardial activation map). We considered three sets of possible root node locations: the low, high and hybrid resolution of the discretisation of the root node parameter space to expose the method's ability to represent root node locations from epicardial activation time map and ECG data modalities.

In the low resolution of the root node discretisation, the possible root node locations were preselected as the centres of each endocardial section following the American Heart Association's segmentation guidelines (Cerqueira Manuel D. et al., 2002), as in Arevalo et al. (2016), while ensuring that any point in either ventricle's endocardia had at least one root node in the same ventricle not more than 2.5 cm away. This selection strategy led to 38 candidate locations (18 in the left and 20 in the right endocardium) in the largest geometry (Mesh-4) and 27 (11 in the left and 16 in the right endocardium) in the smallest one (Mesh-1).

A high resolution of the root node discretisation was also considered by uniformly sampling more root nodes such that the distance from each point to a root node was reduced to 1.5 cm. This high resolution produced 133 candidate root nodes (64 in the left and 69 in the right endocardium) for the largest anatomy and 98 (39 in the left and 59 in the right endocardium) for the smallest one.

Finally, we considered a hybrid resolution that combined the low resolution for the right ventricle with the high resolution for the left ventricle since the latter is presumed to have a stronger influence on the QRS. We anticipate the inference from epicardial activation maps will work better from the symmetric root node distributions (low and high) than from asymmetric ones (hybrid) as the epicardial activation map equally represents both ventricles. On the other hand, the hybrid resolution should improve the inference from 12-lead ECGs since it emphasises the exploration of the root nodes on the left ventricle, which is known to have more influence on the ECG than the right ventricle given its larger myocardial-mass volume.

These candidate root node locations were considered a binary parameter, with the inference yielding ‘in use’ or ‘not in use’ to obtain a good match between simulated and target electrocardiographic signals. We define the range of the number of root nodes as [6, 10] as Cardone-Noott et al. (2016) demonstrated that seven root nodes were sufficient to simulate healthy QRS complexes. We considered no region or ventricle specific rules for the placement of the root nodes to allow for the straightforward extension of the method to disease conditions, such as bundle branch block.

Finally, the conduction speeds were assigned to be within predefined physiological ranges (Durrer et al., 1970). The endocardial and myocardial speeds were bounded within the ranges [100, 200] and [25, 90] cm/s, respectively. Furthermore, we constrained the fibre-directed speed to be larger than the sheet-directed speed and the sheet-directed speed to be larger than the sheet-normal speed to be consistent with the findings presented by Caldwell et al. (2009).

### 2.6 Parameter inference with SMC-ABC method

The combination of conduction speeds and root nodes creates an inference problem with continuous and discrete mixed-type parameter space that challenges many parameter inference algorithms. We propose an SMC-ABC-based algorithm (Appendix A.5) (Drovandi and Pettitt, 2011; Sisson et al., 2007) to efficiently explore our mixed-type parameter space.

In a nutshell, SMC-ABC defines its intermediate distributions (SMC) as approximate posteriors with a series of decreasing cut-off discrepancy values (ABC). In our context, SMC-ABC serves as a parameter-set-based optimisation approach that solves a sequence of simplified optimisation problems (each more manageable than the original one) where each informs the next.

As reviewed in the description of Fig. 1, SMC-ABC uses a population of models with different parameter-sets (similarly to Britton et al. (2013)) that represent the parameter search space of interest. The method then shrinks this parameter space of interest at each iteration, emphasising the ‘promising regions’. This resampling (Step-5 in Fig. 1) is done by replacing the parameter-sets with the highest (12.5%) discrepancies with parameter-sets (mutations or copies) with currently acceptable discrepancy values (Appendix A.5 provides further details on our SMC-ABC algorithm and our strategy for dealing with mixed-type parameter spaces).

The SMC-ABC algorithm's iterative process considers that the current population is a solution to the inference problem when all discrepancies are smaller than a positive tolerance (Appendix A.5). This tolerance threshold can be set according to what level of discrepancy is judged to be acceptable. Here, we hypothesise that the analysis of the inferred population of parameter-sets will highlight the method's representation limit, for example, identifying differences in the position of the root nodes that are negligible from the algorithm's perspective. Therefore, we define a relatively small tolerance (on the scale of the distortion from applying white Gaussian noise-contamination with 20 dB signal-to-noise ratio) to investigate how accurately the method can recover the activation properties from different data modalities.

However, a too-small tolerance will lead SMC-ABC to converge to a single parameter-set, whereas our inference problem is known to have non-unique solutions. Thus, we defined a second stopping criterion relative to the percentage of duplicate parameter-sets to terminate the inference before the population collapses into a single parameter-set. In other words, this second stopping criterion informs the algorithm to terminate when the MCMC process fails to find new acceptable parameter-sets too many times (right dotted arrow in Fig. 1 blue box – bottom left), causing 50% of the parameter-sets in the population to be duplications. Fulfilling either stopping criterion will suffice to terminate the inference process.

### 2.7 Error metrics and post-processing

We propose a k-means based root node aggregation strategy to overcome the performance cap determined by the discretisation of the root node parameter space. More precisely, we define the resulting root nodes locations to be the centroids of the clusters found by applying k-means clustering to the values in each population of parameter-sets. We initialised the centroids in k-means as the most frequently occurring configuration for the root nodes in the population to ensure that the starting configuration was spread out and accounted for a representative number of root nodes. This aggregation strategy allows visualising the inferred root nodes directly in the shared biventricular anatomy. We also report the mean plus-minus (±) standard deviation of the (unsigned) distance between each target root node and the closest centroid from k-means and the mean ± standard deviation of the absolute error in the number of root nodes.

We aggregate the inferred conduction speeds as the median of each speed in the population of parameter-sets. To illustrate the error, we propose a speed-normalised error metric so that we can jointly represent errors from different virtual subjects. This error was defined as

(1) $$error=100*\frac{S^{\prime }-S}{S},\;\left(1\right)$$

where $S^{\prime }$ is the inferred conduction speed value, and S is the ground truth speed.

We adopted the mean ± standard deviation of Pearson's correlation coefficient (Bear et al., 2018; Schaufelberger et al., 2019; Serinagaoglu Dogrusoz et al., 2019) as a measure of disagreement between our inference predictions and the ‘target data’ (Table 2).

**Table 2:** Prediction accuracy of the inference from bidomain ‘target data’ (mean ± standard deviation of Pearson's correlation coefficients). ATM – epicardial activation time maps; low and high resolution of the root node parameter space's discretisation.

Torso-biventricular anatomy	ATM low	ATM high	12-lead ECG low	12-lead ECG high
Mesh-1	0.85 ± 0.05	0.94 ± 0.03	0.87 ± 0.15	0.92 ± 0.09
Mesh-2	0.86 ± 0.04	0.94 ± 0.03	0.91 ± 0.10	0.90 ± 0.10
Mesh-3	0.83 ± 0.03	0.95 ± 0.02	0.95 ± 0.06	0.96 ± 0.04
Mesh-4	0.91 ± 0.02	0.96 ± 0.01	0.93 ± 0.07	0.94 ± 0.07

### 2.8 Hyperparameter calibration

We calibrated the algorithms presented in this study using general and physiological knowledge and adjusted their values to work in one of the 20 virtual subjects (Mesh-4 with ‘Normal speeds’ from Table 1) using the low resolution for the root node discretisation. The results presented in this study were generated using the same calibration of the hyperparameters regardless of the virtual subject, the modality of the ‘target data’, or the resolution of the root node parameter space's discretisation. Further details on the hyperparameter calibration are provided in Appendix A.7.

### 2.9 Computation and software

For each virtual subject (Section 2.2) in our twenty-subject cohort, we conducted inferences using epicardial activation time maps and 12-lead QRS complexes as the ‘target data’. Moreover, we considered three different resolutions of the root node parameter space (i.e. low, high and hybrid). These combinations defined our 120 inference executions, each repeated three times to demonstrate the results’ consistency.

These inference executions were conducted at Amazon Web Services and the Swiss National Supercomputing Centre. Each inference required about one hour to compute in a virtual machine emulating 18 2nd generation Intel Xeon Scalable Processors.

The inference pipeline was developed in Python/Numpy and can be found in https://github.com/juliacamps/Inference-of-healthy-ventricular-activation-properties. The torso-biventricular meshes are also available under request.

The illustrations featured in this manuscript were created using ParaView software (Ahrens, Geveci, and Law 2005), Python's Matplotlib library (Hunter 2007), and Matlab (MATLAB, 2020).

## 3 Results

### 3.1 Prediction of the activation time maps and ECGs from noise-contaminated ‘target data’

**Fig. 2:**
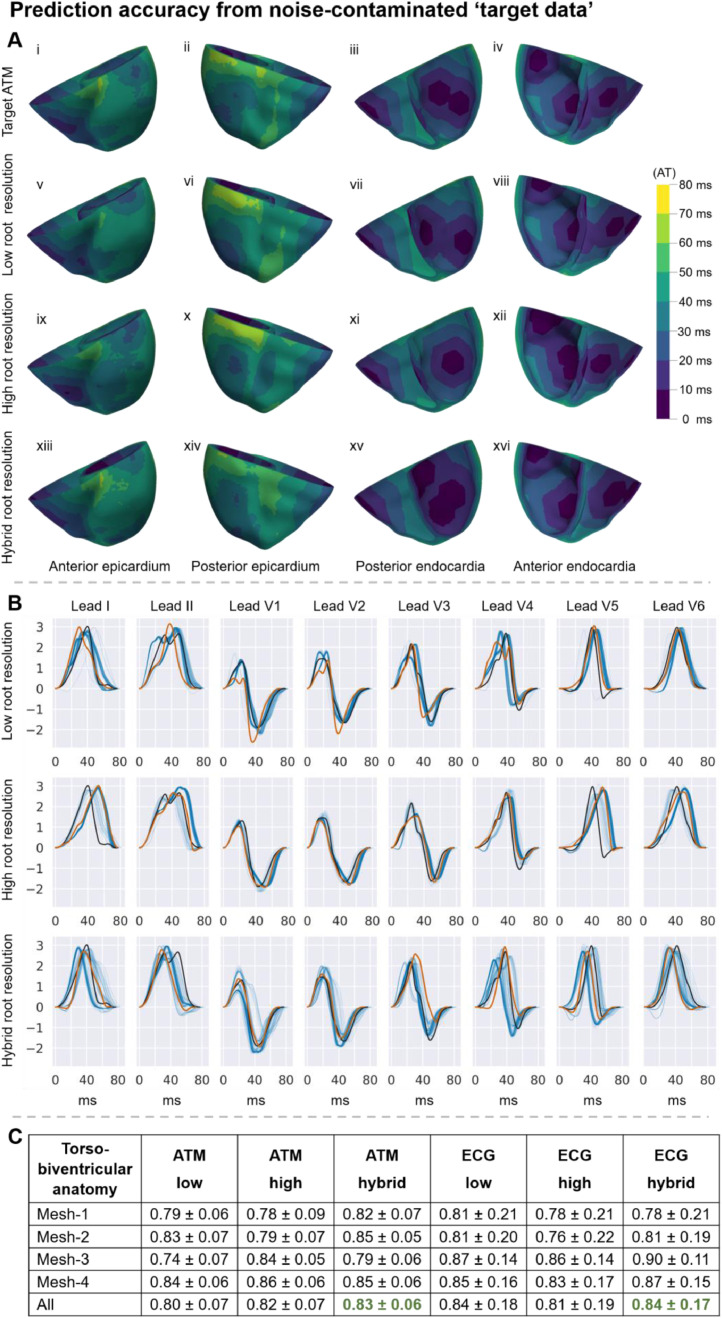
Agreement between predicted and noise-contaminated Eikonal ‘target data’ from the inference process; (A, B) on the virtual subject with Mesh-4 and ‘Slow endocardial speed’ properties (Table 1); (C) mean ± standard deviation of Pearson's correlation coefficients. (A) illustrates three activation time maps (ATM) (one per row) coloured using isochronic activation time (AT) bands of ten ms (colour bar). Top row: target; second row: prediction from the inference using the low resolution for the root node discretisation (0.93 Pearson's correlation coefficient with ‘target data’ – top row); third row: prediction from the inference with high root node resolution (0.89 Pearson's correlation coefficient); fourth row: prediction from the inference with hybrid root node resolution (0.87). Each column shows a different view of the same example-row. (B) shows the ‘target’ (black), ‘predicted population’ (blue) and ‘aggregated solution’ (red) standardised QRSs for the inference guided by 12-lead ECG recordings. Each row accounts for different root node resolutions, from top-to-bottom: low (0.9 and 0.87 for the population-averaged and population-aggregated Pearson's correlation coefficient, respectively), high (0.74 and 0.81), and hybrid (0.77 and 0.83) root node resolutions. Each plot includes the predictions from an evaluation of the inference (512 QRSs) to demonstrate the method's robustness. The amplitude of these standardised QRS signals has no units. (C) mean ± standard deviation of Pearson's correlation coefficients between the predicted and target data. The abbreviations used for this table are ATM – epicardial activation time maps; low, high and hybrid resolution of the discretisation of the root node parameter space.

Fig. 2 illustrates our inference method's ability to replicate the noise-contaminated Eikonal ‘target data’ from epicardial activation maps (A and first half of C) and 12-lead ECGs (B and second half of C).

The inference method accurately replicated the activation maps from noise-contaminated epicardial data (Fig. 2.A and Fig. 2.C), showing slightly greater accuracy for the hybrid root node resolution compared to the low or high resolutions. The replication of the non-septal activation times was more accurate than the septal ones (Fig. 2.A.vii and Fig. 2.A.xv) except for the high-resolution case (Fig. 2.A.xi). Similarly, the replication of the Eikonal 12-lead ECGs was also most accurate when considering the hybrid resolution (Fig. 2.B and Fig. 2.C). Moreover, the DTW-based discrepancy successfully allowed the inference method to replicate all leads with similar accuracies except for lead II that was recovered as monophasic instead of biphasic after the aggregation of the population (Fig. 2.B).

### 3.2 Root node inference from noise-contaminated epicardial activation maps

**Fig. 3:**
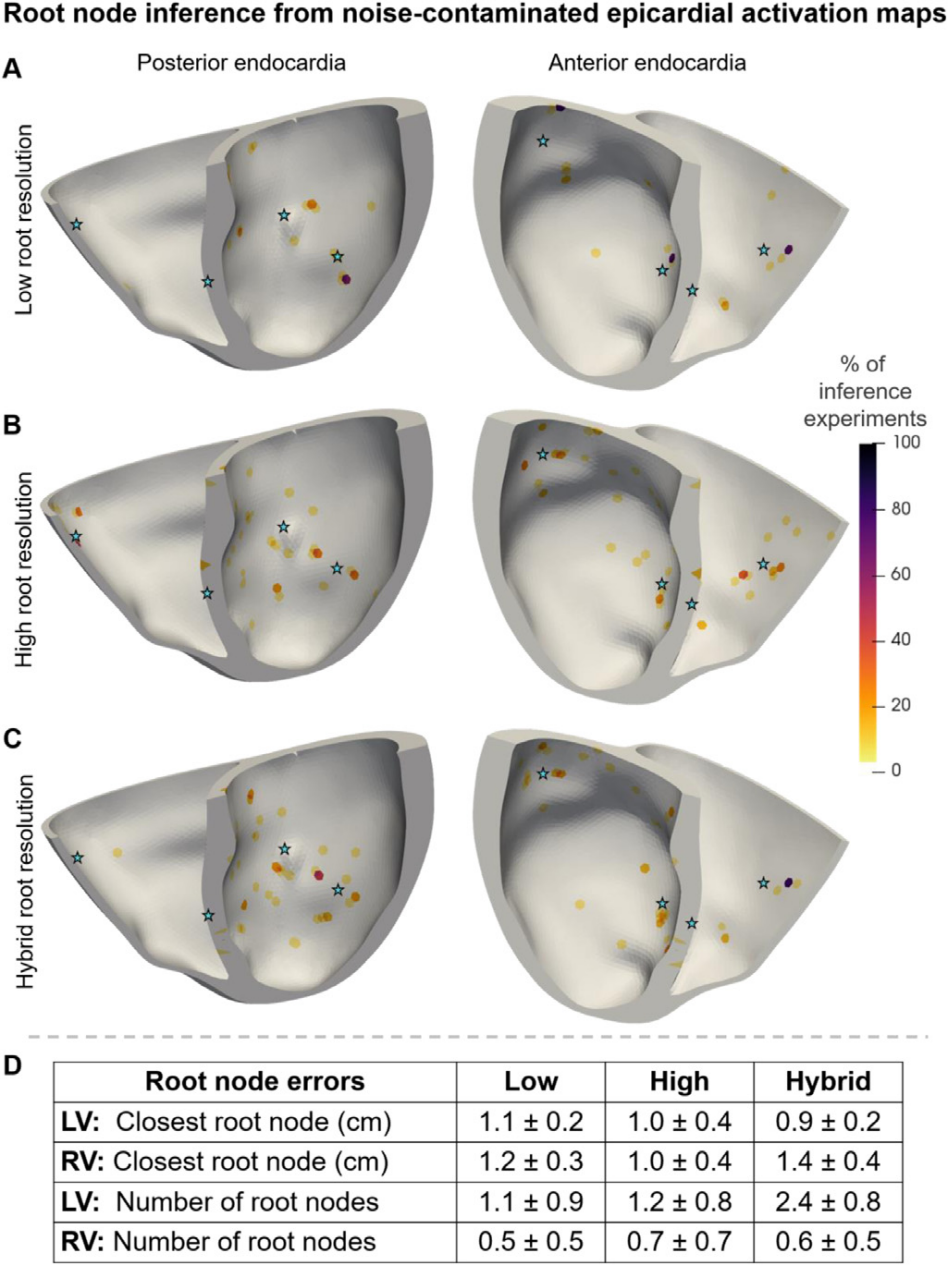
Root nodes inferred from noise-contaminated Eikonal activation maps on Mesh-4 with low (A), high (B), and hybrid (C) resolution of the root node space discretisation and root node inference metrics (Section 2.7) for all meshes (D). The stars indicate the ground truth root node locations. The endocardial surface is coloured as a heatmap showing how often each location was inferred as a percentage. (D) absolute mean ± standard deviation of the distance from the ground truth and error in the number of root nodes inferred from all meshes. LV – left ventricle; RV – right ventricle; low, high, and hybrid resolutions of the discretisation of the root node locations.

Fig. 3 illustrates the inferred root nodes locations from noise-contaminated Eikonal epicardial activation time map ‘target data’ for all virtual subjects with Mesh-4 (Fig. 3.A-C) (see Appendix A.6.1 for Mesh-1, Mesh-2, and Mesh-3), as well as the root node error metrics (Section 2.7) for all anatomies (Fig. 3.D).

Our method inferred more accurately root nodes in non-septal areas since septal activation times are not represented in epicardial maps (Fig. 3.A-C as well as Fig. A.2, Fig. A.3, and Fig. A.4 in Appendix A.6.1). Increasing the resolution of the root node location discretisation aided in recovering root node locations closer to the ground truth from activation maps (Fig. 3.D). The number of root nodes was better identified from the low resolution, whereas increasing the resolution improved the root node positioning accuracy (Fig. 3.D). The hybrid resolution identified better the root nodes in the left ventricle than those in the right ventricle (Fig. 3.D).

### 3.3 Speed inference from noise-contaminated epicardial activation maps

**Fig. 4:**
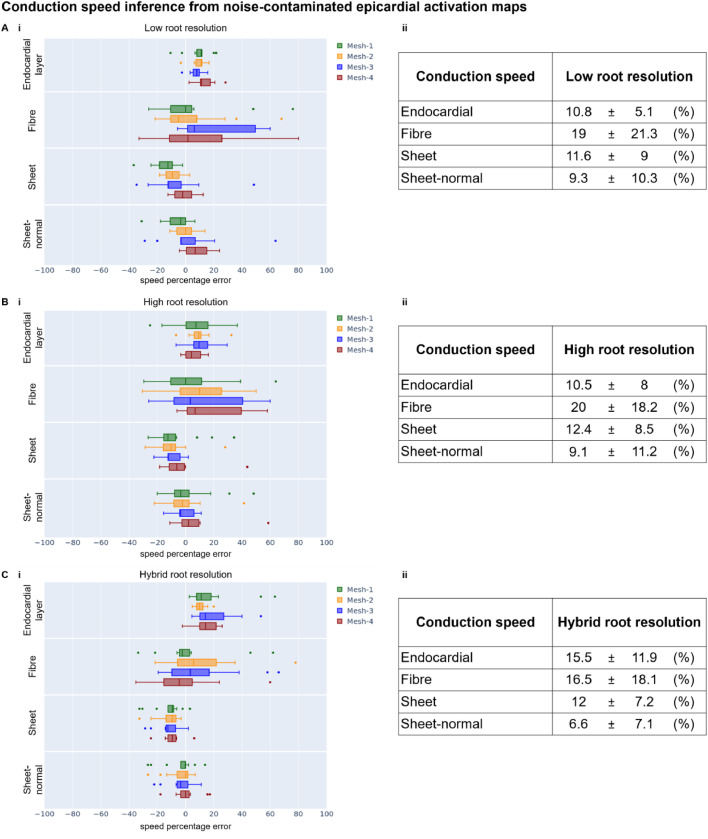
Error in the conduction speeds inference (Eq. (1) in Section 2.7) from noise-contaminated Eikonal epicardial activation maps using low (A), high (B), and hybrid (C) resolution of the root node discretisation. The errors are computed as (Eq. (1)) the percentage over the ground truth conduction speeds (x-axis) represented as (i) box-plots grouped by anatomy (colour) and conduction speed (y-axis); (ii) and represented as absolute mean ± standard deviation.

Fig. 4 reports the errors for the inference of conduction speeds (Eq. (1) in Section 2.7) from noise-contaminated Eikonal epicardial activation time map ‘target data’. The results are shown for low (A), high (B), and hybrid (C) resolution of root node discretisation and grouped by torso-biventricular mesh in the virtual subject (see colour legend in Fig. 4). The right side of the figure provides a numerical summary of the left side to ease readability.

The endocardial and sheet speeds in the inferences implementing the low or high root node resolution were recovered more accurately than the fibre directed speed (Fig. 4.A-B). Throughout all root node resolutions, the endocardial, sheet, and sheet-normal speeds displayed a narrower distribution of error values suggesting that these speeds were consistently recovered at the same accuracy regardless of the characteristics of the virtual subject (Fig. 4).

### 3.4 Root node inference from noise-contaminated ECGs

**Fig. 5:**
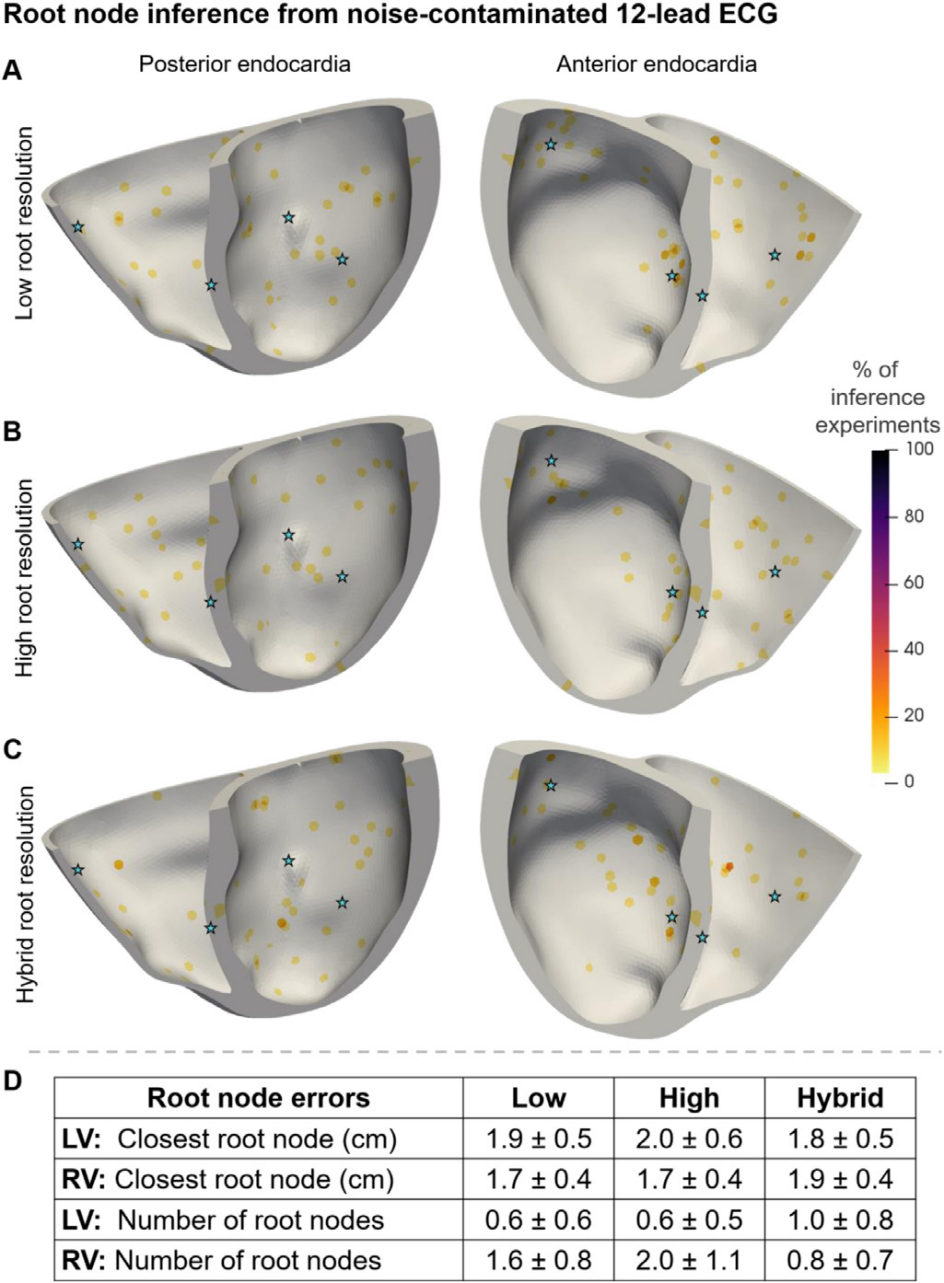
Root nodes inferred from noise-contaminated Eikonal 12-lead ECG on Mesh-4 with low (A), high (B), and hybrid (C) resolution of the root node space discretisation and root node inference metrics (Section 2.7) for all meshes (D). The stars indicate the ground truth root node locations. The endocardial surface is coloured as a heatmap showing how often each location was inferred as a percentage. (D) absolute mean ± standard deviation of the distance from the ground truth and error in the number of root nodes inferred from all meshes. LV – left ventricle; RV – right ventricle; low, high, and hybrid resolutions of the root node locations’ discretisation.

Analogously to Fig. 3, Fig. 5 illustrates the inferred root nodes locations from noise-contaminated Eikonal ECG ‘target data’ for all virtual subjects with Mesh-4 (Fig. 5.A-C) (see Appendix A.6.2 for Mesh-1, Mesh-2, and Mesh-3), as well as the root node error metrics (Section 2.7) for all anatomies (Fig. 5.D).

The inference of root nodes was more accurate on the anterior side of the heart than on the posterior (Fig. 5.A-C as well as Fig. A.5, Fig. A.6, and Fig. A.7 in Appendix A.6.2) due to the left-anterior positioning of the precordial leads in the 12-lead ECG test. Increasing the resolution of the root node discretisation had a little visual effect on the distribution of root nodes identified from ECG (Fig. 5.A-C) compared to epicardial activation maps (Fig. 3.A-C). Moreover, all three resolutions reported similar root node recovery accuracies from ECGs (Fig. 5.D). Overall, the method identified the root nodes better and more concentrated from the Eikonal activation map (Fig. 3.D) than from Eikonal ECG (Fig. 5.D) data.

### 3.5 Speeds inference from noise-contaminated ECGs

**Fig. 6:**
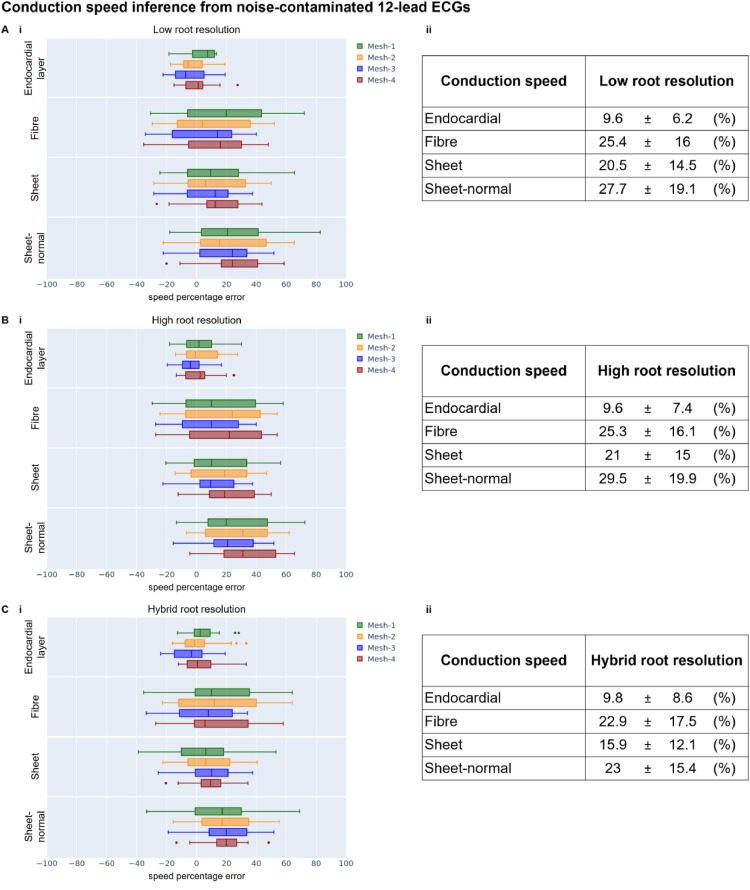
Error in the conduction speeds inference (Eq. (1) in Section 2.7) from noise-contaminated Eikonal 12-lead ECGs for low (A), high (B), and hybrid (C) resolution of the root node discretisation. The errors are computed as (Eq. (1)) the percentage over the ground truth conduction speeds (x-axis) represented as (i) box-plots grouped by anatomy (colour) and conduction speed (y-axis); (ii) and represented as absolute mean ± standard deviation.

Analogously to Fig. 4, Fig. 6 reports the errors for the inference of conduction speeds (Eq. (1) in Section 2.7) from noise-contaminated Eikonal ECG ‘target data’. The results are shown for low (A), high (B), and hybrid (C) resolution of root node discretisation and grouped by torso-biventricular mesh in the virtual subject (see colour legend in Fig. 6). The right side of the figure provides a numerical summary of the left side to ease readability.

Similarly to the inference from the noise-contaminated Eikonal epicardial activation maps (Fig. 4), the conduction speeds inferred from the noise-contaminated Eikonal ECG (Fig. 6) also demonstrated that the endocardial and sheet-directed speeds were recovered more accurately than the fibre and sheet-normal directed speeds. This finding suggests that the endocardial and sheet-directed speeds determined the activation wavefront's propagation speed (Appendix A.8). The endocardial speed was recovered on average more accurately from 12-lead ECG (Fig. 6) than from epicardial activation maps (Fig. 4). The hybrid root node resolution improved the recovery of the conduction speeds from 12-lead ECG (Fig. 6), whereas, as we anticipated (Section 2.5), it played the opposite effect for the inference guided by activation maps (Fig. 4).

### 3.6 Inference from bidomain ‘target data’

To further assess the performance of our inference methodology, bidomain ‘target data’ were generated and used as previously shown for ‘target data’ generated from the eikonal model. Table 2 shows the mean and standard deviation of Pearson's correlation coefficients as a surrogate measure of the inference accuracy to replicate the ‘target data’ generated with bidomain simulations.

Increasing the root node resolution improved the accuracy to infer epicardial activation maps and ECGs generated with bidomain simulations (Table 2). In contrast, the recovery accuracy from noise-contaminated Eikonal ‘target data’ (Fig. 2.C) was unaffected by the resolution of the discretisation of the root nodes.

Table 3 reports the root node inference results from bidomain ‘target data’.

**Table 3:** Root node recovery error metrics (Section 2.7) (absolute mean ± standard deviation) from bidomain ‘target data’. Distance from the ground truth and error in the number of root nodes inferred. LV – left ventricle; RV – right ventricle; ATM – epicardial activation time maps; low and high resolution of the discretisation of the root node parameter space.

Root node errors	ATM low	ATM high	12-lead ECG low	12-lead ECG high
**LV:** Location of closest (cm)	1.1 ± 0.1	0.5 ± 0.0	1.8 ± 0.5	1.6 ± 0.5
**RV:** Location of closest (cm)	0.7 ± 0.1	0.4 ± 0.1	1.5 ± 0.5	1.6 ± 0.6
**LV:** Number of root nodes	0.7 ± 0.7	1.7 ± 0.5	0.7 ± 0.6	0.6 ± 0.9
**RV:** Number of root nodes	0.2 ± 0.2	0.1 ± 0.2	1.5 ± 1.2	0.9 ± 0.9

The high resolution of the root node location discretisation also helped to recover root node locations closer to the ground truth from epicardial activation maps and ECGs (Table 3). However, this increase in resolution was typically accompanied by an overestimation of the number of root node locations in the inference from bidomain epicardial activation maps. In agreement with the results from Eikonal ‘target data’ (Fig. 3.D and Fig. 5.D), the recovery of the root node locations from bidomain data was consistently more accurate from activation maps than from ECGs (Table 3).

Table 4 demonstrates the inference errors for the conduction speed from bidomain ‘target data’.

**Table 4:** Conduction speed recovery error metrics (Eq. (1) in Section 2.7) (absolute mean ± standard deviation) from bidomain ‘target data’. ATM – epicardial activation time maps; low and high resolution of the root node locations’ discretisation.

Conduction speed	ATM low	ATM high	12-lead ECG low	12-lead ECG high
Endocardial	12.9 ± 3.9	2.5 ± 1.8	14.2 ± 14.3	13.9 ± 13.2
Fibre	27.6 ± 8.2	19.0 ± 9.2	28.4 ± 22.7	30.2 ± 24.2
Sheet	5.1 ± 5.6	4.8 ± 2.6	19.5 ± 14.8	14.0 ± 12.6
Sheet-normal	7.4 ± 9.2	5.6 ± 4.7	30.7 ± 13.7	34.1 ± 12.3

**Fig. 7:**
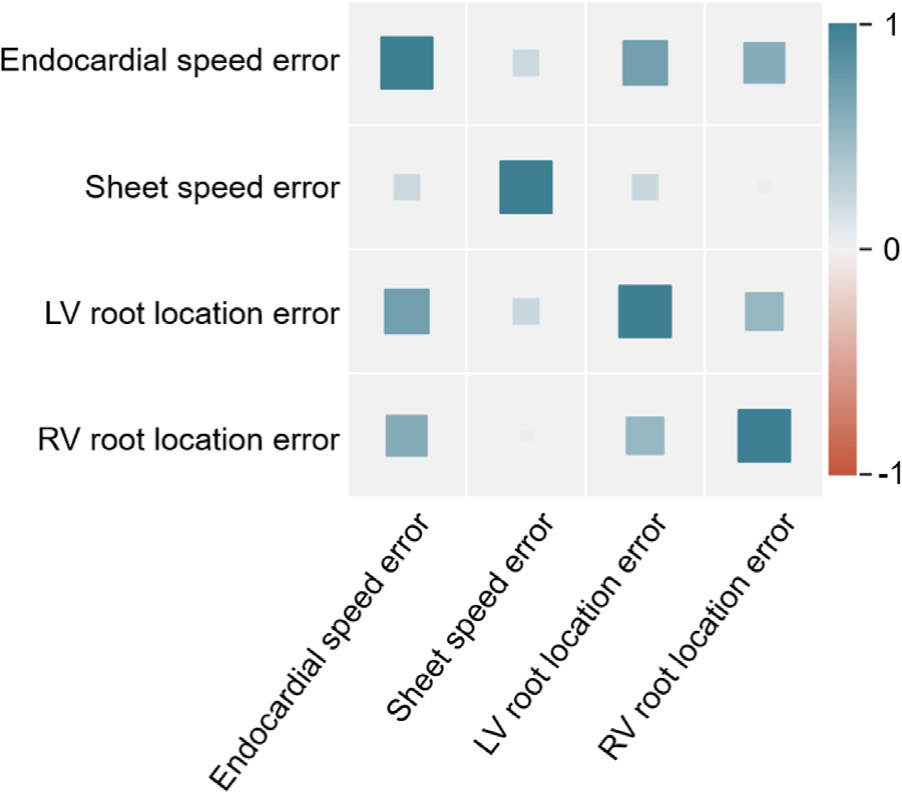
Heatmap of Pearson's correlation coefficients between the root node and the conduction speed inference errors from bidomain simulated epicardial activation time maps.

In agreement with our previous findings (Fig. 4 and Fig. 6), the endocardial and sheet speed were also consistently better recovered from bidomain ‘target data’ in all resolutions and data modalities than the fibre and sheet-normal speeds (Table 4). Moreover, increasing the resolution of the root node discretisation improved the estimation of the conduction speeds (Table 4), as it did for the root nodes (Table 3), suggesting a positive correlation between the identifiability of these activation properties (Fig. 7).

## 4 Discussion

This study presents an inference method combined with CMR-based torso-biventricular Eikonal models to estimate the root nodes and conduction speeds from 12-lead ECG or epicardial activation map data. Our approach aims to serve as an efficient tool for generating cardiac ‘digital twins’, which is of paramount importance for precision cardiology (Corral-Acero et al., 2020). We conducted the simultaneous inference of endocardial, fibre, sheet, and sheet-normal conduction speeds, and the location and number of the root nodes in the endocardium, as these properties determine the activation sequence in the ventricles. The evaluation of the method was conducted on a cohort of twenty virtual subjects to consider the effect of functional (five conduction speed configurations) and anatomical (four anatomies with biventricular myocardial-mass volumes ranging from 74 cm^3^ to 171 cm^3^) variability in the healthy human population on the performance of the inference process. We considered the noise-contaminated simulations as the ground truth for the inferences. The impact of the resolution of root node discretisation on the inference errors was also tested.

In addition to the novel methodology and its ability to simultaneously work with continuous and discrete parameters, the key findings of the study are as follows. Firstly, the method enabled finding populations of models that produced activation sequences with nearly identical electrocardiographic patterns to those observed from the subject's data (Fig. 2) while recovering the root nodes (Fig. 3 and Fig. 5) and the conduction speeds (Fig. 4 and Fig. 6). Secondly, the parameters that define the activation sequence are the root nodes, the endocardial speed, and the sheet-directed speed. Thirdly, each data modality favoured the identifiability of the root nodes in different locations. Namely, epicardial activation maps favoured non-septal root nodes, whereas 12-lead ECG favoured root nodes located on the heart's anterior side.

A key contribution of this study is the development of an inference method to estimate the two types of parameters, namely, the anisotropic conduction speeds (continuous) and the number and locations of the root nodes (discrete) in both ventricles simultaneously. Previous work considered either a known number of root nodes (in simple cases, e.g. paced hearts) or known locations. For example, Giffard-Roisin et al. (2017) estimated the location of two root nodes in the left ventricle and the conduction speeds from ECG data; whereas, Grandits et al. (2020) estimated the activation times of a known set of root nodes and the conduction speeds from epicardial activation maps.

The evaluation of the inference methodology was conducted using synthetic data to know the ground truth values for the ventricular activation properties, as these cannot be measured in healthy subjects. This allowed us to explore the effects of the variability in tissue conduction properties in the healthy human population. We mimicked clinical conditions by contaminating the ‘target data’ using noise to demonstrate the feasibility of our method for clinical environments, and we repeated the experiments using a biophysically detailed model (bidomain) to test the capability of our phenomenological models to perform the inference on realistic data. Moreover, we showed the physiological soundness of our modelling and simulation methods by replicating a clinical electrocardiographic imaging-derived epicardial activation time map (Appendix A.9). This ability to reproduce the physiological patterns demonstrates that our inference methodology is relevant for clinical applications such as the generation of cardiac digital twins (Corral-Acero et al., 2020) towards the realisation of precision medicine.

All our results for the inference of conduction speeds (Fig. 4 and Fig. 6) demonstrated that the endocardial and the sheet speeds were better identified than the fibre and sheet-normal speeds in healthy ventricles in sinus rhythm. When assuming healthy conditions, defined as root nodes in both ventricles and homogeneous tissue-conduction properties, as in Durrer et al. (1970), the endocardial and the sheet-directed speeds (alongside the root nodes) dominated the activation sequence patterns. This difference in the speeds’ identifiability was due to the relatively negligible impact of the fibre and sheet-normal speeds on the activation sequence (Table A.1 in Appendix A.8). These speeds mostly act on the plane parallel to the endocardial layer, governed by a faster isotropic speed (i.e. endocardial speed). Conversely, the sheet directed speed defines the time delay for the endocardial-generated activation patterns to reach the epicardial surface since its orientation is perpendicular to the other speeds. Consequently, under healthy sinus-rhythm conditions, the activation properties that can be inferred from electrocardiographic data were the root nodes, the fast endocardial speed, and the sheet directed speed. However, we hypothesise that the fibre-directed speed will play a more relevant role in pathological conditions, for example, in the presence of slow conductance regions (e.g. scars and fibrosis) or wall thickening (e.g. hypertrophy) since this speed will have time to take over the endocardial patterns.

The inference of the root nodes from epicardial activation maps (Fig. 3, Fig. A.2, Fig. A.3, and Fig. A.4) was better for root nodes in non-septal than in the septal areas. This was because septal root nodes have the least influence on the epicardial activation time map. On the other hand, the inference of root nodes from 12-lead ECGs (Fig. 5, Fig. A.5, Fig. A.6, and Fig. A.7) demonstrated that the anterior locations were better identified than the posterior ones. This asymmetric identifiability of the root nodes manifests the asymmetry of the electrode positioning protocol in the standard 12-lead ECG test. More precisely, each root node has a local effect on the region's activation sequence on the heart that the ECG represents. However, the amplitude of the recorded regional-ECG is inversely proportional to the distance between that region and the electrode, while most electrodes are typically positioned on the left-anterior surface of the torso. Consequently, most changes in the activation sequence patterns in the posterior walls become masked by the electrical activity taking place in the anterior half of the organ for the precordial electrograms.

Overall, we attained higher root node inference accuracies and more consistent locations from using epicardial activation maps (Fig. 3) than from ECG recordings (Fig. 5). The activation map's main advantage is that the data are spatially distributed on a surface (epicardium) that strongly relates to the root node parameter space (except for the septal area). More precisely, the inference from epicardial activation maps can be subdivided into regional sub-problems as local root nodes only influence local patterns in the activation sequence. This phenomenon enables the algorithm to quickly identify partial solutions to the inference problem from epicardial activation maps. On the other hand, all root nodes influence each signal's morphology in the ECG simultaneously.

Our inference results using noiseless bidomain-generated ‘target data’ (Section 3.6) demonstrated that the baseline ability to recover the conduction speeds is conditioned by the identifiability of the root nodes (Fig. 7). In other words, higher resolutions allowed for more accurate root node (Table 3) and speed (Table 4) estimations, highlighting the importance of the choice of resolution as it determines the baseline inference accuracy. These results demonstrate that our inference method generalised to a biophysically detailed source model (bidomain). This, alongside the proof of concept in Fig. A.8 (Appendix A.9), provides evidence of the suitability of our inference method for clinical applications.

### 4.1 Limitations and future work

We implemented the Purkinje network as an isotropic fast endocardial layer with root node locations that emulate the hotspots of Purkinje myocardial junctions. This strategy has been extensively used in previous studies (Cardone-Noott et al., 2016; Lyon et al., 2018; Wallman et al., 2014) and shows equivalent behaviour in the activation maps to that observed from human data reported by Durrer et al. (1970). However, Durrer et al. (1970) also reported differences of about ten milliseconds in the activation between root nodes located two centimetres apart from each other that our modelling strategy has not considered. A possible extension could include a realistic representation of the cardiac conduction system (Grandits et al., 2020; Sebastian et al., 2013), adding additional complexity and uncertainty to the inference problem. Note that the dominance of the endocardial and sheet speeds would prevail despite the inclusion of a Purkinje network, as in Grandits et al. (2020), since the cause of this dominance is the parallel influence of a fast speed in the endocardial plane (e.g. fast endocardial speed or realistic Purkinje network) and the fibre and sheet-normal speeds. Another possible improvement could be to test the implications of using a different fibre orientation model (Bayer et al., 2012; Doste et al., 2019; Rossi et al., 2014).

Overall, this study presented the foundations of a novel pipeline capable of non-invasively calibrating cardiac digital twins for healthy subjects from synthetic epicardial activation maps and 12-lead ECG recordings. Our approach was designed to easily accommodate disease conditions, such as pathological tissue heterogeneities (scars or fibrosis). Moreover, the results using bidomain-generated ‘target data’ (Section 3.6) and clinical ‘target data’ (Appendix A.9) to guide the inference suggest that the presented approach can be directly translated to work on clinical ECG recordings. Moreover, we anticipate that our methodology can be adapted to work with pathological data to generate cardiac ‘digital twins’ in daily clinical practice.

## CRediT authorship contribution statement

**Julia Camps:** Conceptualization, Methodology, Software, Formal analysis, Validation, Writing – original draft, Visualization. **Brodie Lawson:** Methodology, Software, Writing – review & editing. **Christopher Drovandi:** Methodology, Writing – review & editing. **Ana Minchole:** Conceptualization, Writing – review & editing. **Zhinuo Jenny Wang:** Formal analysis, Writing – review & editing, Visualization. **Vicente Grau:** Resources, Writing – review & editing. **Kevin Burrage:** Methodology, Writing – review & editing. **Blanca Rodriguez:** Conceptualization, Formal analysis, Resources, Writing – original draft, Supervision.

## Declaration of Competing Interest

The authors declare that they have no known competing financial interests or personal relationships that could have appeared to influence the work reported in this paper.

## Appendix Supplementary materials

Image, application 1
