# Supplementary material for: Inference of ventricular activation properties from non-invasive electrocardiography
Source: Med Image Anal. 2021 Oct;73:102143. doi: 10.1016/j.media.2021.102143 (PMC8505755; doi:10.1016/j.media.2021.102143)
Supplement: Supplementary file 1 [file mmc1.pdf]

Supplementary Materials: Inference of ventricular activation properties from non-invasive electrocardiography

Julia Camps[1*], Brodie Lawson[2,3], Christopher Drovandi[2,3], Ana Minchole[1], Zhinuo Jenny Wang[1], Vicente Grau[4], Kevin Burrage[1,2], Blanca Rodriguez[1*]

[1]Department of Computer Science, University of Oxford, Oxford, United Kingdom.

[2]Australian Research Council Centre of Excellence for Mathematical and Statistical Frontiers (ACEMS), Queensland University of Technology (QUT), Brisbane, Australia.

[3]QUT Centre for Data Science (CDS), Queensland University of Technology, Brisbane, Australia.

[4]Institute of Biomedical Engineering (IBME), University of Oxford, Oxford, United Kingdom.

*Corresponding author: {julia.camps, blanca}@cs.ox.ac.uk

Appendix A

A.1. Bidomain model

The bidomain model is the gold standard formulation for cardiac electrophysiology simulations. The bidomain assumes that each point in space represents an average between multiple cells and that there are two electrical subspaces in the heart, namely, the intracellular space and the extracellular. The combination of both assumptions implies that at every point in space, we have an intracellular and an extracellular potential, φ_i and φ_e, respectively. Therefore, transmembrane potential, V_m, is defined as $V_m = \varphi_i - \varphi_e$. The current densities, $\bar{J}_i$ and $\bar{J}_e$, in these two spaces are defined as

$$\bar{J}_i = g_{ix}\frac{\partial\varphi_i}{\partial x}\bar{a}_x + g_{iy}\frac{\partial\varphi_i}{\partial y}\bar{a}_y + g_{iz}\frac{\partial\varphi_i}{\partial z}\bar{a}_z$$

$$\bar{J}_e = g_{ex}\frac{\partial\varphi_e}{\partial x}\bar{a}_x + g_{ey}\frac{\partial\varphi_e}{\partial y}\bar{a}_y + g_{ez}\frac{\partial\varphi_e}{\partial z}\bar{a}_z\ ,$$

where $\bar{a}_x$, $\bar{a}_y$, and $\bar{a}_z$ are unit vectors along the x, y, and z fibre-axes, respectively. More precisely, x represents the fibre (same direction as the fibre), y the sheet (perpendicular direction to the fibre, but along the same tissue sheet), and z the sheet-normal (normal to the tissue sheet plane) fibre-directions. Each fibre-direction has an associated conductivity, g. Thus, g_x, g_y, and g_z are the conductivities along each of the fibre-directions. Moreover, these conductivities can vary between the intracellular and extracellular domains.

The bidomain model imposes the conservation-of-current constraint such that any current leaving one subspace must enter the other one. Thus, we can write $-\nabla\cdot\bar{J}_i = \nabla\cdot\bar{J}_e = I_v$, where I_v is the transmembrane current per unit volume.

Ohm's law, $\bar{J} = \sigma\bar{E}$, defines a current density, $\bar{J}$, as the product of an electrical field, $\bar{E}$, and conductivities, σ. Furthermore, assuming that the magnetic field is not time-varying, through Faraday's law, we can redefine the electrical field in terms of the electrical potential, $\bar{E} = -\nabla\varphi$. By applying both laws, we can rewrite the current densities as $\bar{J}_i = -\sigma_i\nabla\varphi_i$ and $\bar{J}_e = -\sigma_e\nabla\varphi_e$.

Next, by applying the conservation of currents assumption that relates the two domains through the cells' membranes, we can rewrite the density currents in terms of the transmembrane current, $\bar{I}_m$, as follows

$$\beta I_m = \nabla \cdot (\sigma_i \nabla\varphi_i)$$
$$\beta I_m = -\nabla \cdot (\sigma_e \nabla\varphi_e)\,,$$

where β accounts for the membrane surface to volume ratio.

Moreover, by employing the capacitance formulation, the transmembrane current can be written as $I_m = C_m \frac{\partial V_m}{\partial t} + I_{ion}(V_m, \eta)$, where C_m is the cell's membrane capacitance, I_{ion} is the ionic current that leaks in and out of the cell due to its mechanisms, and η is the set of variables of the cell model. These ionic currents are governed by a set of non-linear differential equations that will vary for each cell model implementation.

Finally, the bidomain model is

$$\nabla \cdot (\sigma_i \nabla\varphi_i) = \beta\left(I_m = C_m \frac{\partial V_m}{\partial t} + I_{ion}(V_m, \eta)\right)$$
$$\nabla \cdot (\sigma_e \nabla\varphi_e) = -\beta\left(I_m = C_m \frac{\partial V_m}{\partial t} + I_{ion}(V_m, \eta)\right).$$

A.2. Electrical propagation Eikonal model

The bidomain equations provide a reaction-diffusion-based biologically detailed description of the electrical propagation in the human heart by assuming two electrical subspaces, namely the intracellular and the extracellular space. Computation of bidomain simulations is costly; thus, simplifications such as the Eikonal equation have been proposed to study the mechanisms defining the cardiac activation sequence (Cedilnik & Sermesant, 2020; Colli Franzone et al., 1990; Wallman et al., 2012).

The Eikonal model converts simulation of electrical wave propagation in a cardiac mesh into a shortest-path-finding problem. The Eikonal method produces equivalent activation sequences to bidomain or monodomain models for only a fraction (up to three orders of magnitude lower) of the computational cost (Wallman et al., 2012). Moreover, the Eikonal method can simulate propagation on coarse meshes, whereas the bidomain approach has convergence restrictions on mesh resolution. For these reasons, we use the Eikonal model to generate activation map data in our inference method of the human ventricular activation properties.

The basic formulation of the Eikonal equation is $\sqrt{\nabla d^T \cdot \nabla d} = 1$, where d is a distance vector. However, we can rewrite the Eikonal equation to account for an electrical wavefront propagation time in an anisotropic cardiac mesh (Colli Franzone et al., 1990). Namely, we apply $\nabla d = v\nabla t$, where v is speed, $v(f, s, n)$, where f, s and n are orthonormal vectors that account for the fibre, sheet (transmural) and sheet-normal directions, respectively; and where t is the travelling time $t(x, y, z)$

passing through a point (x, y, z), so our formulation is $\sqrt{\nabla t^T \cdot V \cdot \nabla t} = 1$, with $V = \begin{bmatrix} x_l & x_t & x_n \\ y_l & y_t & y_n \\ z_l & z_t & z_n \end{bmatrix} \begin{bmatrix} v_f^2 & 0 & 0 \\ 0 & v_s^2 & 0 \\ 0 & 0 & v_n^2 \end{bmatrix} \begin{bmatrix} x_l & x_t & x_n \\ y_l & y_t & y_n \\ z_l & z_t & z_n \end{bmatrix}^T$, where v_f , v_s , and v_n are the speeds in the fibre, sheet, and sheet-normal directed speeds, and where $[x_i, y_i, z_i]$ defines the vector $\vec{\imath}$, where i can be either f, s, or n.

We implement the Eikonal equation by interpreting the tetrahedral-mesh as a connected graph where electric current can go directly from two connected nodes, a and b, through an existing edge (a, b), where the distance between a and b is the Euclidean distance, namely, $\left\|\overrightarrow{(a, b)}\right\|_2$. Therefore, from the Eikonal approach, we have that the time cost between two adjacent nodes a and b is $c_{a,b} = \sqrt{\overrightarrow{(a, b)}^T \cdot V^{-1} \cdot \overrightarrow{(a, b)}}$. Finally, we can solve the Eikonal's activation times using a multisource-multidestination extension of Dijkstra's algorithm (Dijkstra, 1959). Wallman et al. (2012) demonstrated that our edge-constrained implementation yields equivalent results to the fast marching method (Konukoglu et al., 2007) while being significantly faster to compute. We set the root node locations as starting sites with $t = 0$ and all the other nodes of the biventricular mesh as destinations for which we want to calculate t.

A.3. Virtual Subjects

This section provides further details on how the conduction speeds considered to design our cohort of 20 virtual subjects link to the conductivities employed by Mincholé et al. (2019).

We considered three fast-endocardial speeds to account for a slow (120 cm/s), normal (150 cm/s), and fast (179 cm/s) Purkinje network, and two myocardial speed scenarios, normal and fast. The normal myocardial conduction speeds (50, 32, and 29 cm/s for the fibre, sheet, and sheet-normal speeds, respectively) were chosen to match the [extracellular, intracellular] conductivity pairs used in the bidomain model, namely [5.46, 1.5], [2.03, 0.45], and [2.03, 0.225] millisiemens/centimetre (mS/cm) in the fibre, sheet and sheet-normal directions, respectively. Similarly, the fast myocardial conduction speeds (88, 49, and 45 cm/s) corresponded to conductivities [10.92, 3], [4.06, 0.9], and [4.06, 0.45] mS/cm. The root node locations were set to seven homologous root node locations (three in the right and four in the left ventricle) following Cardone-Noott et al. (2016) on root node configurations that produced realistic healthy ECG recordings.

Hereafter, the simulation protocol for the bidomain-generated data considered as the ground truth for this inference study is outlined as described in the Supplementary Material of Mincholé et al. (2019). The tetrahedral element edge lengths were set to 0.04 cm (re-meshed to ensure numerical convergence of the finite element solvers), compared to the coarser (re-meshed) geometries used in the Eikonal models, which had edge lengths ranging from 0.15 to 0.23 cm. The finer resolution of the meshes used for the bidomain simulations implied that the number of elements was 31 million compared to the 65000 in the coarser version of the mesh for the Eikonal. The activation maps produced in an equivalent configuration for the bidomain model and the Eikonal model present differences due to the lower resolution (edge-length) and stronger discretisation (edge-constrained shortest path-finding algorithm) characterised in the implementation of the Eikonal models compared to the bidomain models. These differences are manifested as a 'patchy' or 'pixelated' activation map compared to the bidomain's, which are smoother. This 'patchiness' translates into noise-like artefacts in the ECG requiring additional filtering; thus, distortion, compared to the equivalent bidomain-produced signals.

The bidomain simulations were conducted using Chaste software (Pitt-Francis et al., 2009), an extension of the O'Hara-Rudy action potential model (O'Hara et al., 2011) proposed by Dutta et al. (2017). Moreover, the bidomain models implemented the following electrophysiological heterogeneities:

- They accounted for physiological apex-to-base action potential duration differences. These were modelled as a gradual decrease of the potassium ionic conductance (I_{Ks}) (from apex to base), resulting in differences of 40 ms in the action potential duration.
- Transmural heterogeneities were modelled as three regions of cellular models: the endocardial region from endocardium to 45% of the transmural width, the mid-myocardial region from 45% to 70% transmural width), and the epicardial region from 70% to the epicardial surface. Then, the cell models' action potential properties in these regions were modelled as suggested by O'Hara et al. (2011). These gradients resulted in interventricular action potential duration differences of 25 ms between left and right ventricles.

Note that these heterogeneities mainly affect the cardiac cycle's repolarisation phase; thus, the Eikonal model without the heterogeneities can still reproduce a similar QRS complex.

The torso geometries of these virtual subjects served to inform the positions of the electrodes according to the standard 12-lead ECG electrode positioning protocol, as in Cardone-Noott et al. (2016) and Mincholé et al. (2019). These virtual electrode positions on the torso allowed simulating 12-lead ECG recordings.

A.4. Computation of the ECG from the activation time map

The QRS complexes in specific ECG leads were computed from the activation time maps simulated through the Eikonal model using the pseudo-ECG equation (Gima & Rudy, 2002), as in Mincholé et al. (2019). Alternative biophysically detailed approaches exist. For example, Poste (2018) presents an accurate ECG computation approach for reaction-diffusion cardiac models, and Pezzuto et al. (2017) presents another ECG computation approach for fast phenomenological cardiac models, such as the Eikonal model. However, the cost of these approaches represented a computational bottleneck for our inference pipeline. Moreover, as illustrated in Fig. A.1, the simplest and fastest pseudo-ECG approach accurately reproduces the ECGs computed from the bidomain. Therefore, we opted for an even simpler and faster pseudo-ECG approach proposed in Gima and Rudy (2002). This formulation combined with step functions, as in Potyagaylo et al. (2014), and a discretisation of the Eikonal formulation (see Section A.2) enables an even faster sparse implementation of the pseudo-ECG equation that only updates the amplitude values with the elements showing changes in the gradients of their potential.

The pseudo-ECG equation for a 1D fibre source at a given electrode location, (x', y', z'), takes the form

$$\Phi_e(x', y', z') = \frac{a^2 \sigma_i}{4\sigma_e} \int (-\nabla V_m) \cdot \left[\nabla \frac{1}{r}\right] dx\, dy\, dz\,,$$

where V_m is the transmembrane potential, $\nabla V_m$is its spatial gradient, r is the Euclidean distance from a given point (x, y, z) to the electrode's location, a is a constant that depends on the fibre's radius, and σ_i and σ_e are the intracellular and extracellular conductivities, respectively. An ECG signal is obtained by then considering this integral throughout the ventricular activation sequence period.

Working from an activation map generated by the Eikonal approach, there is no cell model or time course data for V_m. However, a QRS complex can still be produced (within a constant factor) by merely

assigning V_m to be one or zero, depending on whether the node in question has been activated. The pseudo-ECG algorithm cannot recover the exact amplitude of the ECG leads, as it does not take into account the different impedances and attenuations between each source (x, y, z) and sensor (x', y', z') points pair. Moreover, we know that the organ conductivities in the torso modulate the amplitude of the ECG signals. Consequently, we may affect the signals' amplitude by considering a homogenous bath and directly projecting the ECG to the electrode location. This phenomenon could be addressed by considering a discrepancy metric that relies on the ECG signals' derivatives (Ramírez et al., 2017). However, we chose to standardise the target and predicted ECG signals to generate an interpretable visualisation to monitor the inference process. The standardisation process normalises the ECG signals to have zero mean and one standard deviation. This assumption allows us to ignore constants in the original formulation, such as a, and rewrite the pseudo-ECG equation as a sum over all mesh elements,

$$\Phi_e(x', y', z') = \sum_{j=1}^{N_{src}} -(\nabla V_m)_j \cdot \left[\nabla \frac{s_j}{r_j}\right],$$

where N_{src} is the number of tetrahedral source elements, $(\nabla V_m)_j$ is an estimated gradient over the jth tetrahedral element and s_j its normalised volume. Distances r_j are calculated using the centroids of each element. We estimate these gradients by assigning $V_{mi} = 1$ if the node i is activated, and $V_{mi} = 0$ if it is not, which is sufficient to generate a scaled-amplitude ECG signal.

Fig. A.1 illustrates a comparison of the amplitude-standardised versions of the ECG recording obtained from using a diffusion model (blue) from the epicardium to the torso surface compared to the pseudo-ECG algorithm (green).

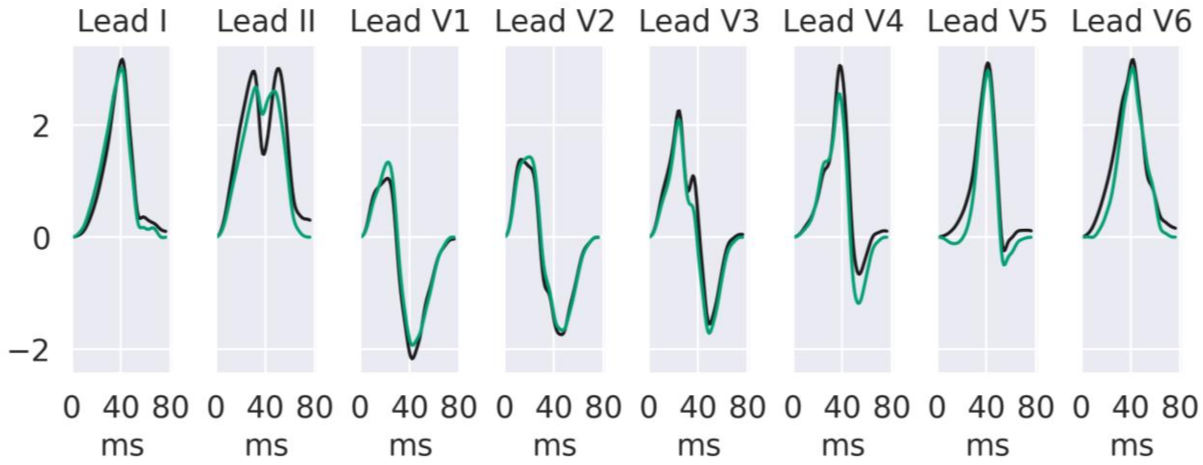

Fig. A.1. ECG calculation justification example. ECG calculated as the diffusion from the bidomain-generated map to the electrodes (black). ECG calculated by the pseudo-ECG equation on the bidomain-generated activation time map (green). The amplitude of these signals is standardised to have mean zero and standard deviation one (y-axis). The time units are ms (x-axis).

Fig. A.1 demonstrates that the pseudo-ECG equation produces realistic ECG-QRS recordings from the Eikonal model that match the diffusion-based signals.

For the 12-lead ECG, we consider the eight independent leads. In other words, we ignore lead III and the augmented leads since they are linear combinations of other leads. Furthermore, the cost of solving the Eikonal formulation in a fine mesh (31 million elements) is 25 min in one CPU, which would be too long, whereas simulating coarse meshes (65000 elements) can be done in 1.6 s. Hence coarse

meshes are a necessary evil for the efficient generation of cardiac digital twins. However, simulating ECG signals from coarse meshes produces noise-like artefacts (Potse & Kuijpers, 2010; Schuler et al., 2019; Tate et al., 2019). Thus, we filter the ECG recordings with a lowpass filter with a cut-off frequency set to 150 Hz following the Nyquist-Shannon sampling theorem and the guidelines in frequential information in the ECG from Sörnmo and Laguna (2005). Finally, the signals are aligned to start at 0 standardised voltage.

A.5. Novel SMC-ABC algorithm for mixed-type parameter space

Sequential Monte Carlo - approximate Bayesian computation (SMC-ABC) integrates the ideas behind SMC and ABC algorithms. SMC discovers regions of interest in the parameter space by starting from a simpler version of the original sampling problem and gradually reintroducing its complexity. SMC evolves a population of parameter-sets through a series of intermediary distributions. The current population of parameter-sets informs each mutation step, which modifies the population to satisfy the next sampling problem. This process results in a high degree of exploration initially, with each intermediate distribution trading some exploration for more exploitation in promising regions of the parameter space (similarly to simulated annealing [Lew et al. 2009]).

ABC targets an approximate posterior for model parameters θ (parameter-set values), $p(\theta|\rho(d, d_{pred}) \leq \epsilon)$. Here $\rho(d, d_{pred})$ is a measure of discrepancy between the target data d, and d_{pred}, the predicted data from a parameter-set θ; and ϵ is the cut-off discrepancy, with $\epsilon = 0$ recovering the true posterior. ABC defines a small discrepancy tolerance since the target data is sometimes impossible to reproduce due to noise (the discrepancies we used are described in Section 2.4).

A real-life example of this strategy would be to search for a small object in a low-resolution image; the method would scan the image, identify the region where the object cannot be and blur it to enable increasing the resolution of the other areas. After a few iterations, the image would have a few focal areas of high resolution, and all the rest would be blurred.

As outlined in Step-5 from Fig. 1, the SMC approach consists of a resampling step that replaces low-quality parameter-sets (parameter-sets with high discrepancy) and then a mutation step that recovers parameter-set uniqueness. As ABC does not define a likelihood, resampling is achieved simply by splitting the current parameter-sets into 'to keep' and 'to replace' groups according to their discrepancy value and the current cut-off discrepancy. Then the algorithm replaces the 'to replace' group with an equivalent number of parameter-sets copied from the 'to keep' group selected at random. The copied parameter-sets are then mutated by repeatedly implementing small changes whilst ensuring that their discrepancies remain below the smallest discrepancy in the 'to replace' set. Finally, the SMC-ABC accepts/rejects these proposed mutations according to an approximated Metropolis-Hastings ratio, as in the Markov chain Monte Carlo (Gilks, 2005). As we are seeking to identify parameter-sets with low discrepancy and not specifically targeting samples from a Bayesian posterior, such an approximation can be used without issue.

Our implementation of SMC-ABC accounts for a mixed-type (continuous and discrete) parameter space. We now describe the details of the method.

In generating new proposals for SMC-ABC's mutation steps (substep 4 in Step-5 from Fig. 1), we ignore any dependencies between the discrete (root node locations) and continuous (conduction speeds) parameter spaces. We use the typical random Gaussian jumps approach informed by the current covariance of the parameter-sets for the continuous space. However, for the discrete root node space, we propose a novel strategy to generate proposals independent of a parameter-set's current values

but informed by the whole population. Independent proposal distributions are asymmetric but can significantly accelerate SMC approaches by being more likely to suggest large jumps across the parameter space (South et al., 2019).

More precisely, we first determine the number of root node locations for a new parameter-set. To choose the number of root nodes, 80% of the time, we inherit the number of root nodes from a random parameter-set in the population. To prevent premature convergence, the remaining 20% of the time, we sample the number of root nodes from a normal distribution with a mean of eight and a standard deviation of one (Cardone-Noott et al., 2016) over the range defined for the number of root nodes, rounding to the nearest integer (Section 2.5). Next, we place a Dirichlet prior (i.e. multivariate Beta distribution) on the propensity that a candidate root node location is 'in use'. We treat the frequency of a candidate location being 'in use' in our population as a sample from a multinomial distribution. This treatment choice allows us to calculate these propensities from the current set of parameter-sets as the Dirichlet distribution is the conjugate prior for a multinomial likelihood. Due to conjugate priors' properties, our posterior on propensities for the candidate root node locations will also be Dirichlet distributed. Given the number of root nodes to be 'in use', we select them one at a time by first sampling from the Dirichlet posterior implied by the 'relevant' parameter-sets in the population to obtain a set of propensities. Then we sample from the (single-trial) multinomial distribution with these propensities.

A parameter-set is 'relevant' if it has the same number of root nodes as the parameter-set being created and the same root node locations 'in use' as those selected so far during this process. The population of relevant parameter-sets that informs selecting the next candidate root node location becomes smaller after each selection. Consequently, the first few selections of root nodes will represent the existing patterns in the population of parameter-sets, while the final selections should explore new candidate root node locations. Importantly, by taking this approach and only using matching parameter-sets to update the propensities each time a candidate root node is selected, we capture interdependencies between root node locations in the population.

A.6. Root node inference results from noise-contaminated (Eikonal) 'target data'

This section reports the visualisation of the root node inference results from noise-contaminated Eikonal 'target data' from the virtual subjects with anatomies Mesh-1, Mesh-2, and Mesh-3, which were not featured in the main manuscript.

A.6.1. Root node inference from noise-contaminated epicardial activation maps

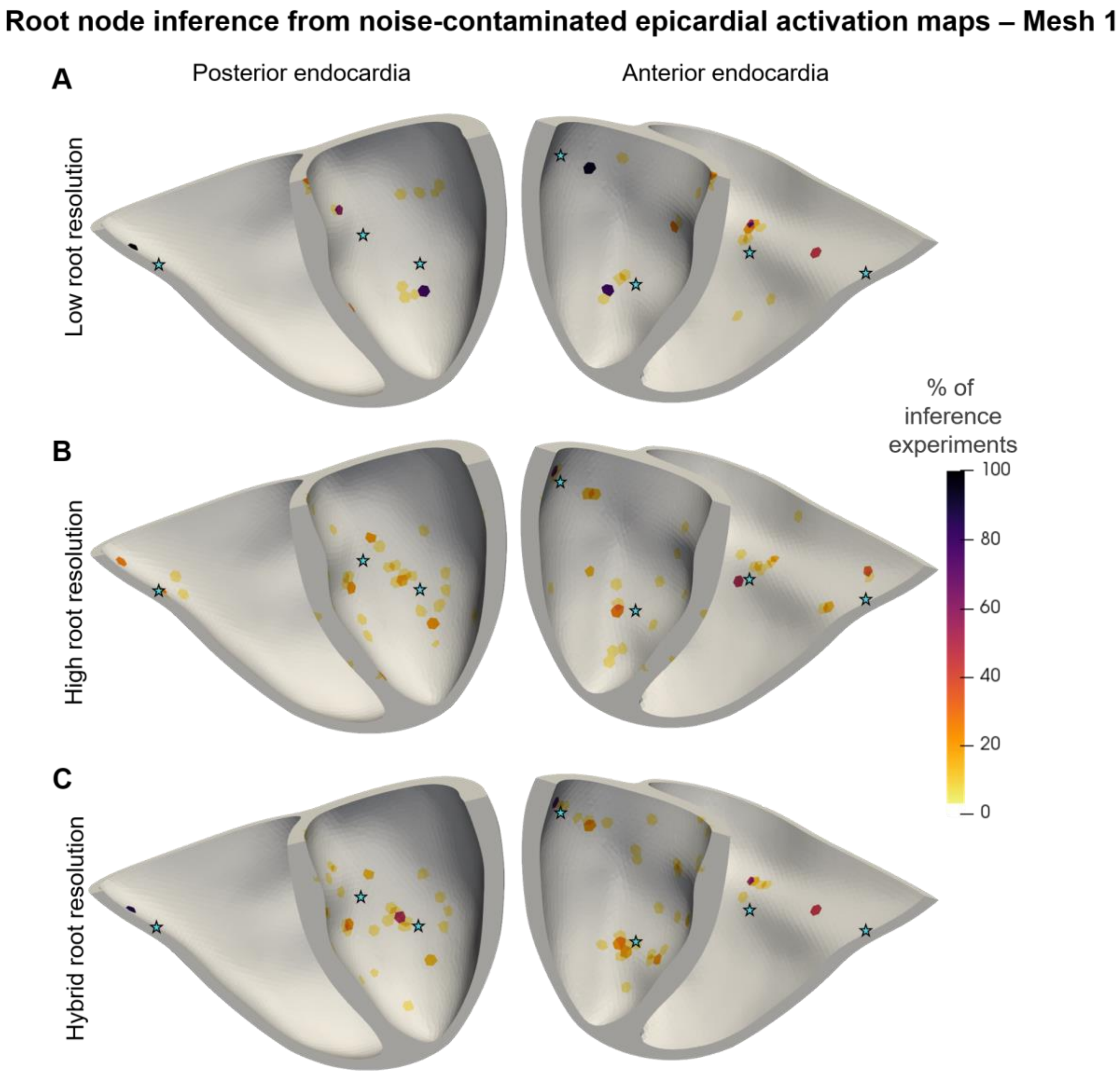

*Fig. A.2. Root nodes inferred from noise-contaminated Eikonal activation maps on Mesh-1 with low (**A**), high (**B**), and hybrid (**C**) resolution of the root node space discretisation. The stars indicate the ground truth root node locations. The endocardial surface is coloured as a heatmap showing how often each location was inferred as a percentage.*

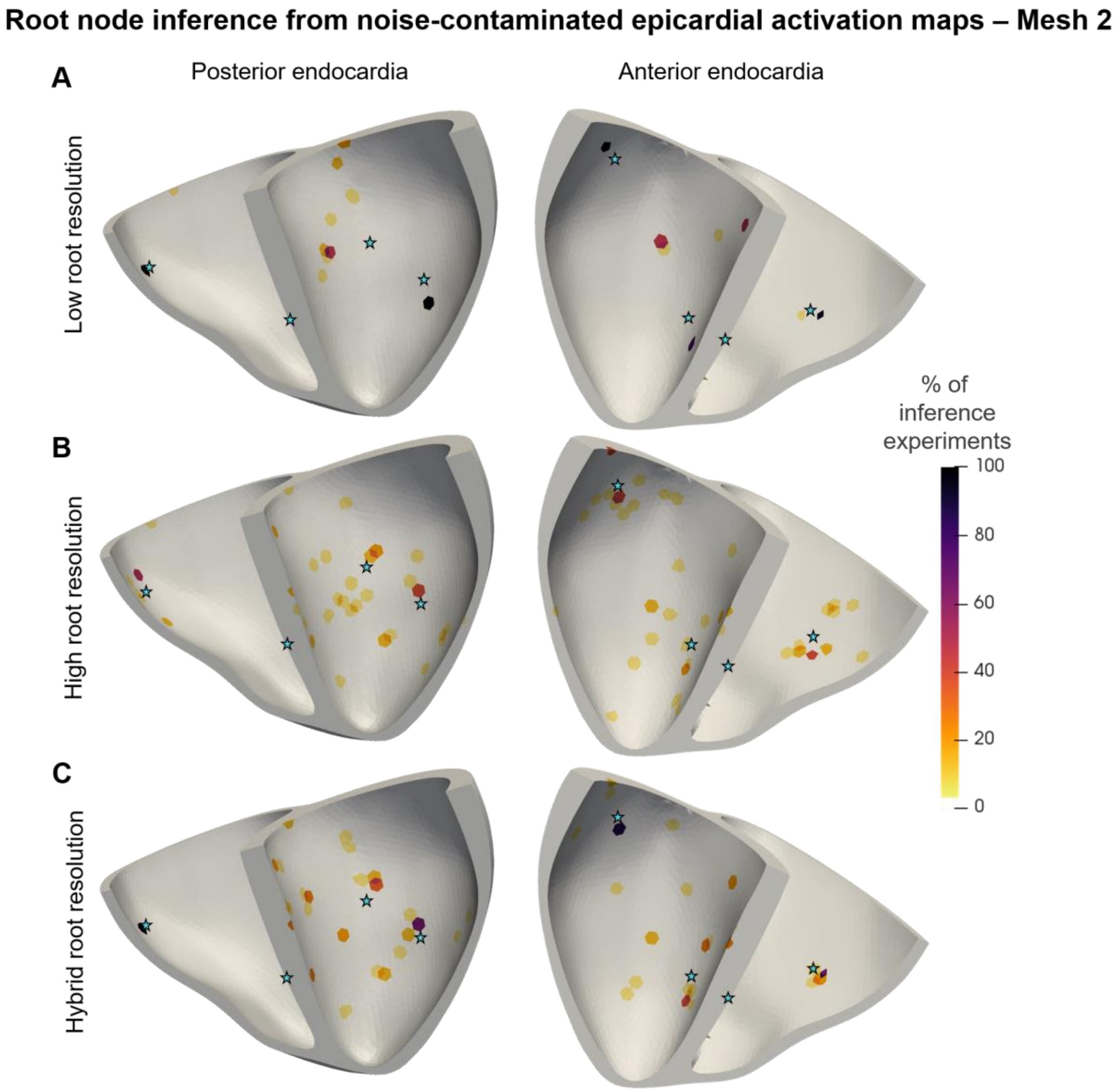

*Fig. A.3. Root nodes inferred from noise-contaminated Eikonal activation maps on Mesh-2 with low (**A**), high (**B**), and hybrid (**C**) resolution of the root node space discretisation. The stars indicate the ground truth root node locations. The endocardial surface is coloured as a heatmap showing how often each location was inferred as a percentage.*

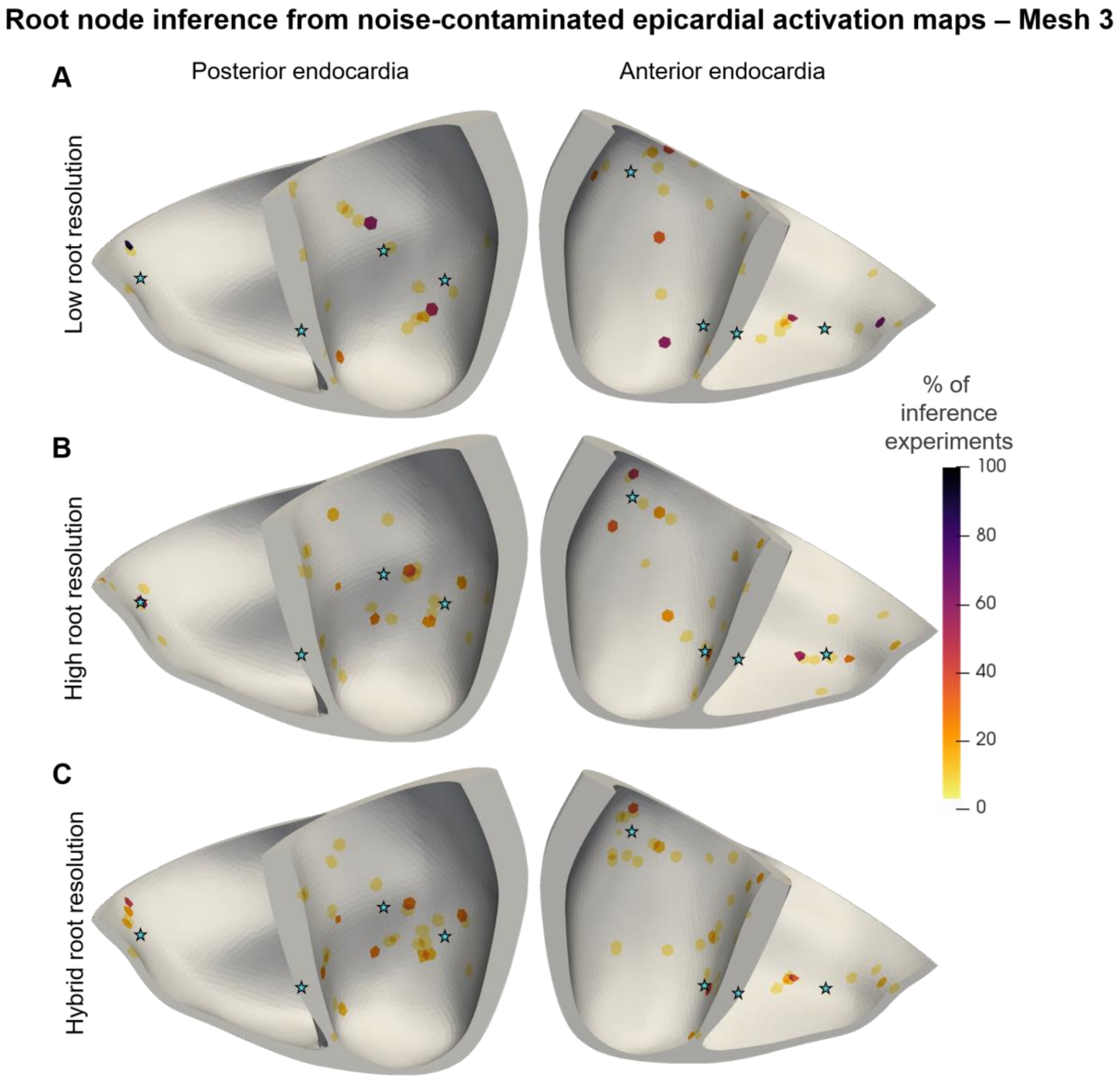

*Fig. A.4. Root nodes inferred from noise-contaminated Eikonal activation maps on Mesh-3 with low (**A**), high (**B**), and hybrid (**C**) resolution of the root node space discretisation. The stars indicate the ground truth root node locations. The endocardial surface is coloured as a heatmap showing how often each location was inferred as a percentage.*

In agreement with the results from Mesh-4 (Fig. 3), the root node inference from noise-contaminated Eikonal epicardial activation maps considering Mesh-1 (Fig. A.2), Mesh-2 (Fig. A.3), and Mesh-3 (Fig. A.4) were more accurate in the non-septal regions of the heart and were overall most accurate for the high resolution of the discretisation of the root node parameter space.

A.6.2. Root node inference from noise-contaminated ECGs

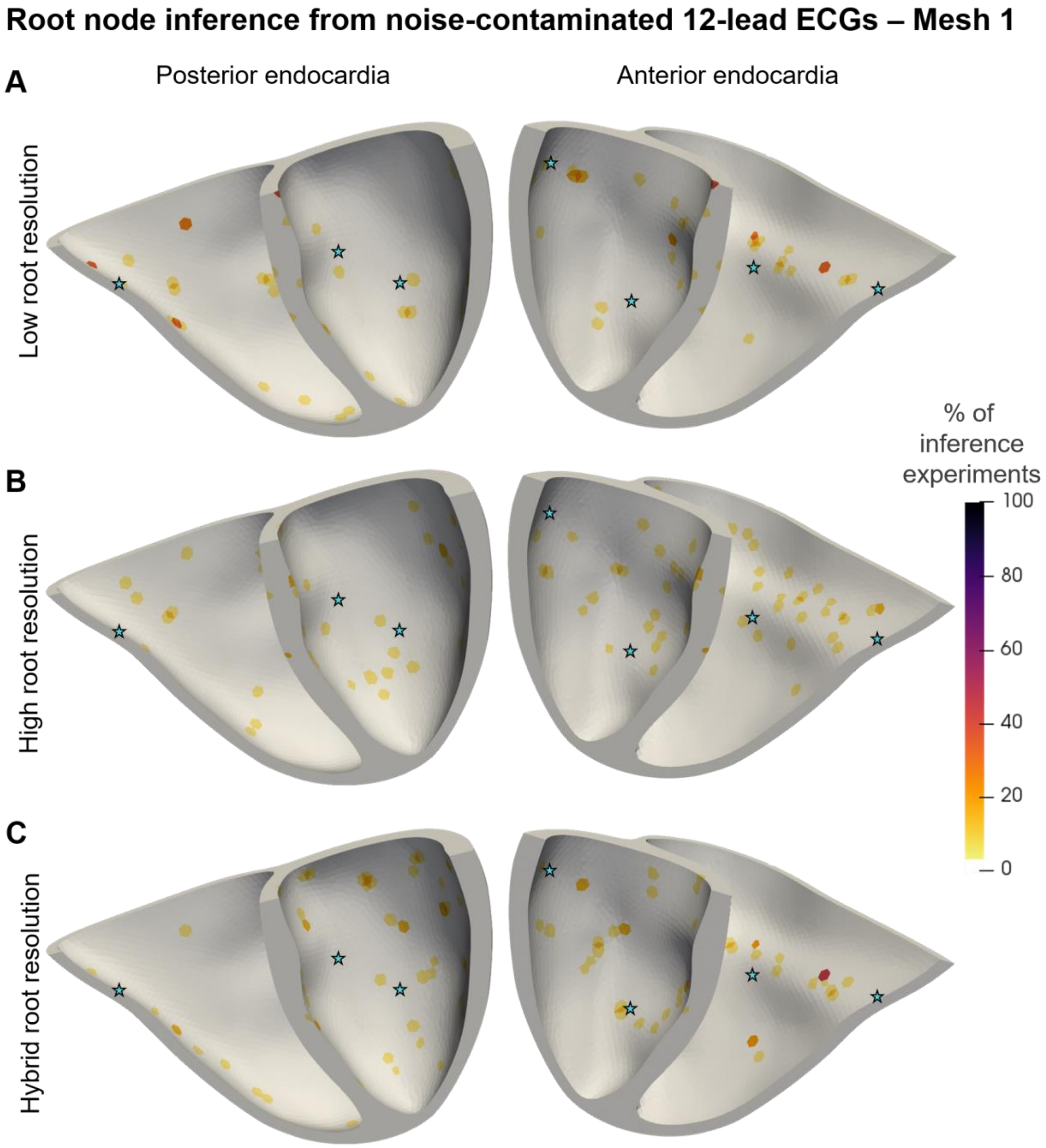

*Fig. A.5. Root nodes inferred from noise-contaminated Eikonal 12-lead ECG on Mesh-1 with low (**A**), high (**B**), and hybrid (**C**) resolution of the root node space discretisation. The stars indicate the ground truth root node locations. The endocardial surface is coloured as a heatmap showing how often each location was inferred as a percentage.*

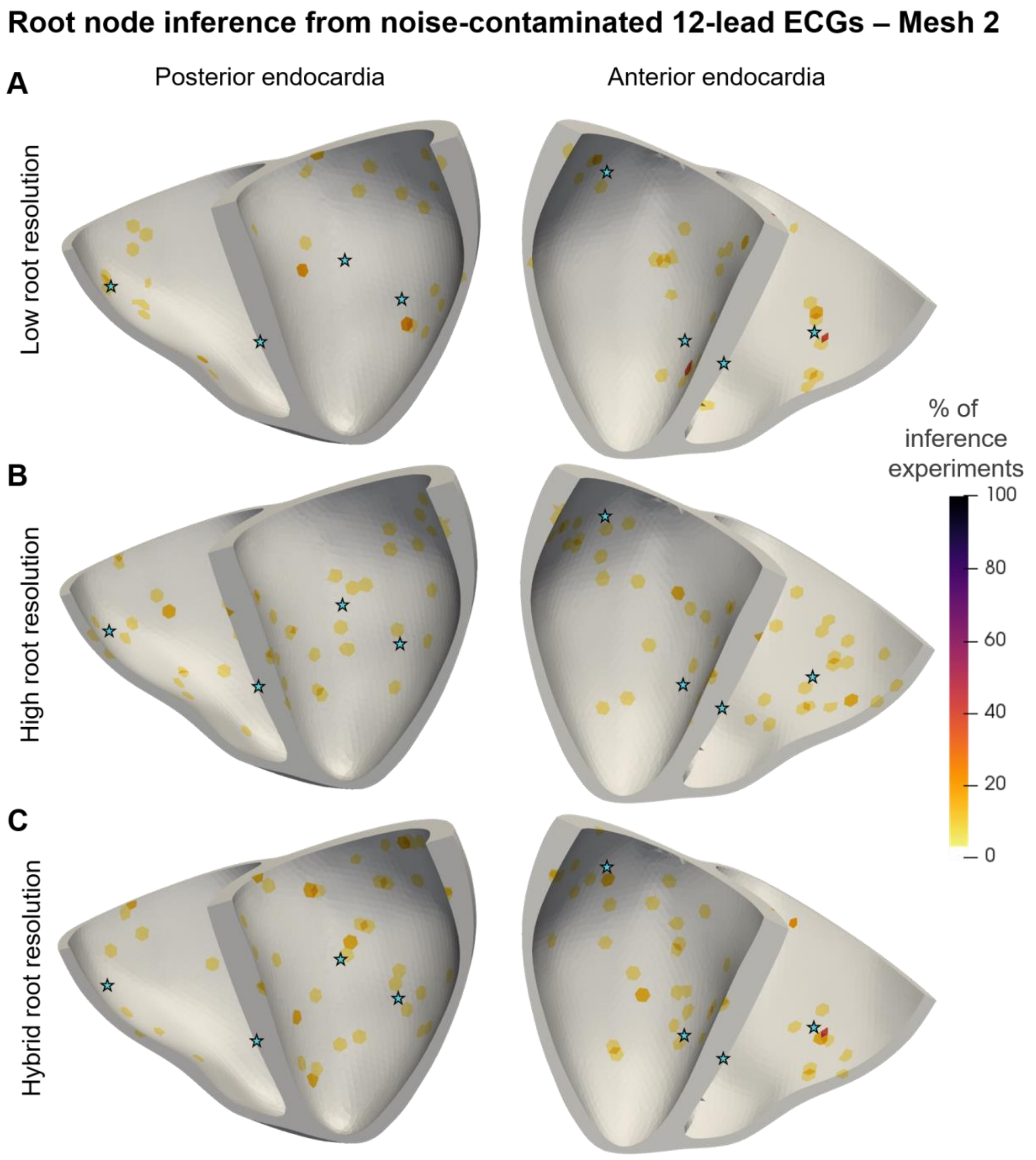

*Fig. A.6. Root nodes inferred from noise-contaminated Eikonal 12-lead ECG on Mesh-2 with low (**A**), high (**B**), and hybrid (**C**) resolution of the root node space discretisation. The stars indicate the ground truth root node locations. The endocardial surface is coloured as a heatmap showing how often each location was inferred as a percentage.*

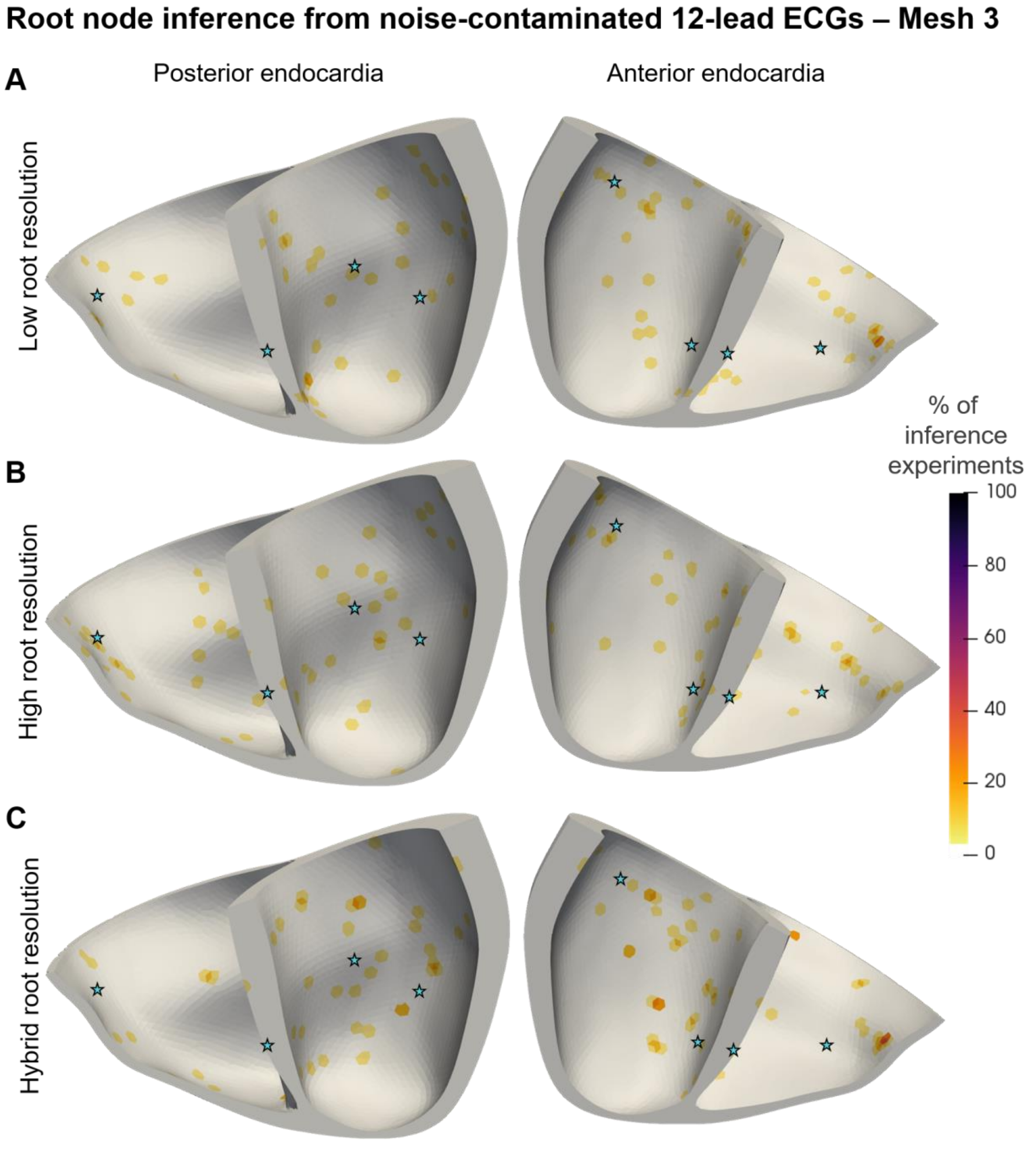

*Fig. A.7. Root nodes inferred from noise-contaminated Eikonal 12-lead ECG on Mesh-3 with low (**A**), high (**B**), and hybrid (**C**) resolution of the root node space discretisation. The stars indicate the ground truth root node locations. The endocardial surface is coloured as a heatmap showing how often each location was inferred as a percentage.*

Also, in agreement with the results from Mesh-4 (Fig. 5), the root node inference from noise-contaminated Eikonal ECG on the other torso-biventricular anatomies (Fig. A.5, Fig. A.6, and Fig. A.7) estimated the locations on the anterior side of the heart more accurately than in the posterior side, and, overall, less accurately than from Eikonal epicardial activation maps (Fig. A.2, Fig. A.3, and Fig. A.4).

A.7. Hyperparameter values

This section describes the hyperparameters and the rationale for the values chosen for them.

A.7.1. Stopping criteria hyperparameters

- Desired positive tolerance: 0.01 (discrepancy units).
  The inverse electrocardiographic problem can have multiple solutions. Thus, we define a non-zero tolerance to halt the inference if the population is formed by distinct solutions to the problem instead of forcing the method to converge to the best solution. This stopping criterion is essential for clinically relevant applications since it aids the model to maintain the set of solutions to the inverse problem and, thus, be more informative for the user. However, in this study, we defined a purposely small value to ensure that the method identified some solutions and provided a baseline for future studies on appropriate values for this hyperparameter. This will strongly depend on the modality of the data and the discrepancy metric adopted.
- Stopping 'duplicates' of parameter-sets: 50%.
  There may not be a 'diverse' population of parameter-sets that satisfied our small discrepancy tolerance. Thus, we defined a 'duplicates' threshold to terminate the inference process before all solutions collapsed into one. More precisely, we hypothesise that the problem will have multiple solutions from which one will be slightly better due to the modelling strategy, discrepancy metric, and noise-realisation. Consequently, we want to ensure that the inference process is terminated before losing the population's diversity once the method cannot find new solutions to the inverse problem.

A.7.2. SMC-ABC hyperparameters

- Maximum number of MCMC steps: 100.
  This parameter prevents a single iteration from deciding to perform more than 100 MCMC steps, which would happen when the estimated information is low.
- Retain ratio (used to calculate alpha in the Metropolis-Hastings ratio): 0.5.
  This parameter balances the preference of replacing duplicate parameter-sets that are underrepresented for slight modifications of better-represented parameter-sets.
- Population size: 512 parameter sets.
  The population size should be sufficient to represent the regions sparsely in the parameter space to enable the subsequent identification of local minima composing the multiple solutions to the inverse problem. We explored values that are powers of 2 (e.g. 128, 256, 512 and 1024) and observed that increasing further from 512 did not add diversity when solving the inference problem in our calibration virtual subject.
- Mutation step size: 12.5% of the parameter-sets.
  The mutation step size is a compromise hyperparameter since it will improve the quality of the exploration to choose a small value at the cost of slower convergence.
- Use-population ratio to determine the number of root nodes: 80%.
  80% of the times, the new parameter-set inherits the number of root nodes from a random parameter-set and 20% of the times chooses a random value from the prior distribution of the number of root nodes. These values were chosen to maintain the search of values close to seven, as suggested by Cardone-Noott et al. (2016).
  We want the algorithm to rely on the values it converges towards; however, we need to explore new root node counts continuously.

A.7.3. Latin hypercube sampling hyperparameters

The endocardial and fibre-directed conduction speeds were assigned within predefined physiological ranges (Durrer et al., 1970). The two remaining speeds were sampled as proportionality factors to guarantee that the sheet and sheet-normal speeds were spread out while fulfilling the constraint that the sheet speed should be slower than the fibre speed and that the sheet-normal speed should be slower than the sheet speed. The sheet speeds were sampled as a proportion of the fibre speed, and the sheet-normal speed as a proportion of the sheet speed. Thus, the sheet speed value was obtained from the sampled proportionality value multiplied by the sampled value for the fibre speed and analogously for the sheet-normal speed.

- The sheet-directed speeds proportionalities to the fibre-directed speeds were sampled uniformly across the range [0.2 – 1].
- Analogously, the sheet-normal-directed speeds proportionalities to the sheet-directed speeds were sampled uniformly across the range [0.6 – 1].

These proportionality factors and ranges ensured that the resulting sampled speeds would be within human physiological ranges.

A.8. Evaluation of the effect of the conduction speeds in the Eikonal formulation with a fast endocardial layer

Table A.1 reports the Pearson's correlation coefficient between variations of the same calibration to illustrate the impact of modifying each speed individually in the Eikonal model when modelling a healthy heart with a fast isotropic endocardial layer speed. The reference activation map was modelled using the 'normal speeds' configuration from Table 1, namely, the healthy configuration of the root nodes suggested by Cardone-Noott et al. (2016) and the following conduction speeds: 150 cm/s, 50 cm/s, 32 cm/s, and 29 cm/s for the endocardial, fibre-directed, sheet-directed, and sheet-normal directed speeds, respectively.

**Torso-biventricular anatomy**	**Endo x 2**	**Endo / 2**	**Fibre x 2**	**Fibre / 2**	**Sheet x 2**	**Sheet /2**	**Normal x 2**	**Normal / 2**
Mesh-1	0.921	0.93	0.994	0.995	0.966	0.951	0.997	0.996
Mesh-2	0.922	0.93	0.995	0.995	0.964	0.949	0.996	0.995
Mesh-3	0.93	0.921	0.996	0.996	0.959	0.953	0.996	0.996
Mesh-4	0.929	0.924	0.997	0.998	0.954	0.947	0.998	0.998

Table A.1. Pearson's correlation coefficients between activation maps from different speeds. The correlation coefficient between the baseline 'normal speeds' (Table 1), namely, 150 cm/s, 50 cm/s, 32 cm/s, and 29 cm/s for the endocardial, fibre-directed, sheet-directed, and sheet-normal directed speeds, respectively, and a simulation of the same calibration with one speed changed. Each row displays the results for one of our four torso-biventricular anatomies, and each column displays the correlation coefficients when modifying the indicated speed. Endo x 2: twice the endocardial speed (300 cm/s). Endo / 2: half the endocardial speed (150 cm/s). Fibre x 2: twice the fibre-directed speed (100 cm/s). Fibre / 2: half the fibre-directed speed (25 cm/s). Sheet x 2: twice the sheet-directed speed (64 cm/s). Sheet / 2: half the sheet-directed speed (16 cm/s). Normal x 2: twice the sheet-normal directed speed (58 cm/s). Normal / 2: half the sheet-normal directed speed (14.5 cm/s).

The impact on the simulated epicardial activation map of changing the value of a conduction speed in the Eikonal model was significantly higher for the endocardial and sheet-directed speeds than for the fibre and sheet-normal speeds (Table A.1).

A.9. Reproducing real clinical data

Fig. A.8 demonstrates our inference method's ability to replicate patterns observed in clinical data.

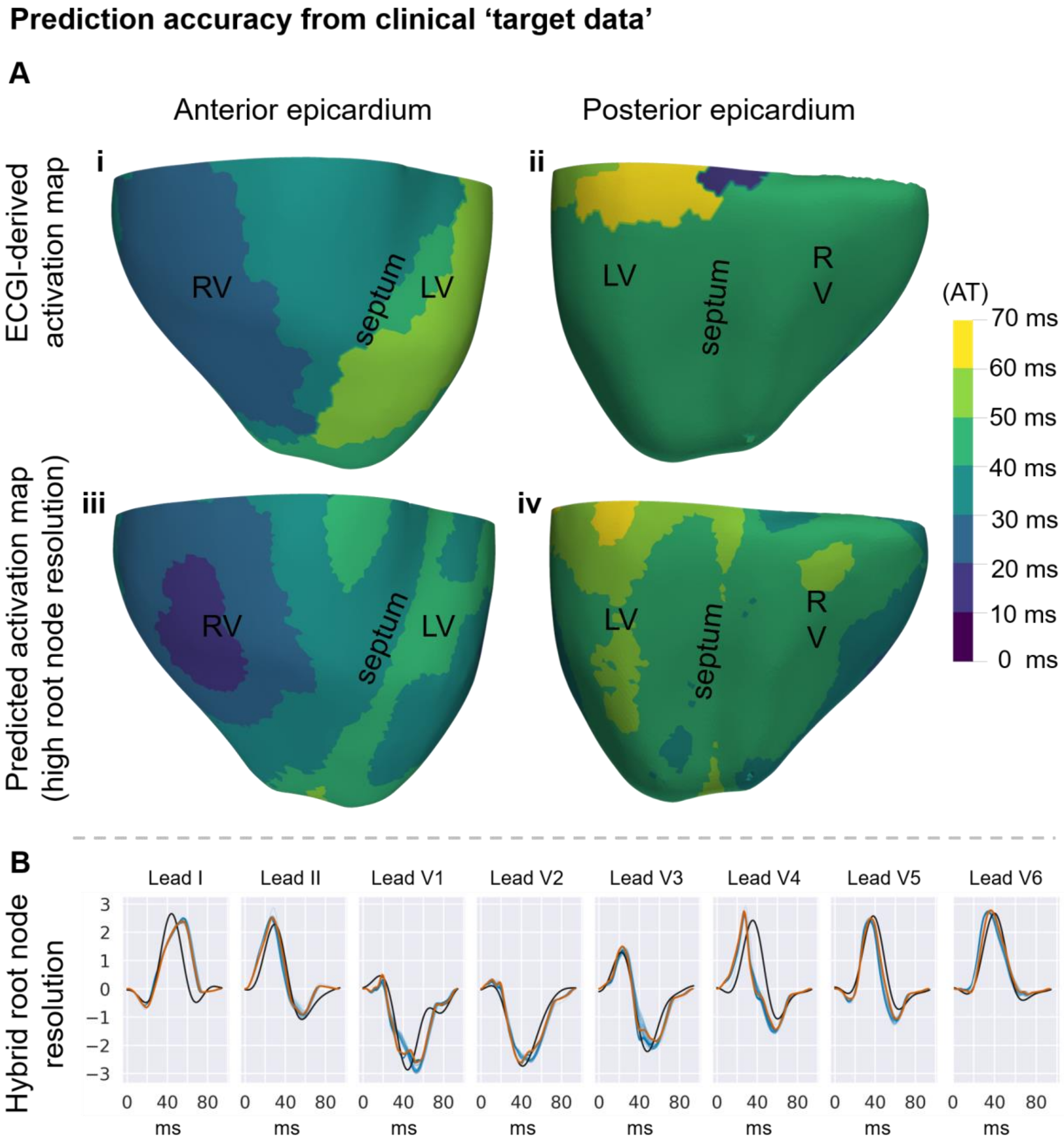

*Fig. A.8. Reproduction of clinical data. (**A**) Activation time map predicted from the inference method (**A**.iii-iv) from the electrocardiographic imaging-derived epicardial activation time (AT) map as target (**A**.i-ii) (using the minimum derivative of the potentials as the activation times) acquired from a healthy subject presented in* Andrews et al. *(2019). (**B**) shows the 'target' (black), 'predicted population' (blue) and 'aggregated solution' (red) standardised QRSs for the inference guided by clinical 12-lead ECG recordings. These target signals correspond to the averaged clinical QRS complexes from the original subject corresponding to Mesh-4. The inference used the hybrid root node resolution (0.88 and 0.87 for the population-averaged and population-aggregated Pearson's correlation coefficient, respectively). Each plot includes the predictions from an evaluation of the inference (512 QRSs) to demonstrate the method's robustness. The amplitude of these standardised QRS signals has no units.*

Our inference method, paired with patient-specific meshes, demonstrated being capable of reproducing activation sequences consistent with those observed from the patients' electrocardiographic imaging solutions (Fig. A.8.A). The Eikonal simulation (Fig. A.8.A.iii) successfully

replicated the epicardial wave breakthroughs observed from the electrocardiographic imaging solution (Fig. A.8.A.i), as well as other patterns in the ECGI-derived activation map.

Our inference method also accurately replicated the clinical 12-lead ECG-QRSs (Fig. A.8.B) from the subject corresponding to Mesh-4. The conduction speeds inferred from these QRS complexes were 110, 69, 51 and 51 cm/s for the endocardial, fibre, sheet and sheet-normal speeds. These values are all within physiologically healthy values reported from human experimental measurements (Durrer et al., 1970). Similarly, the root node locations inferred for this healthy subject presented no pathological abnormalities.

This ability to reproduce the physiological patterns in the activation maps demonstrates that our inference methodology is relevant for applications such as the generation of cardiac digital twins (Corral-Acero et al., 2020) towards the realisation of precision medicine.

Andrews Christopher, Cupps Brian P., Pasque Michael K., & Rudy Yoram. (2019). Electromechanics of the Normal Human Heart In Situ. *Circulation: Arrhythmia and Electrophysiology*, *12*(11), e007484. https://doi.org/10.1161/CIRCEP.119.007484

Cardone-Noott, L., Bueno-Orovio, A., Mincholé, A., Zemzemi, N., & Rodriguez, B. (2016). Human ventricular activation sequence and the simulation of the electrocardiographic QRS complex and its variability in healthy and intraventricular block conditions. *EP Europace*, *18*(suppl_4), iv4–iv15. https://doi.org/10.1093/europace/euw346

Cedilnik, N., & Sermesant, M. (2020). Eikonal Model Personalisation Using Invasive Data to Predict Cardiac Resynchronisation Therapy Electrophysiological Response. In M. Pop, M. Sermesant, O. Camara, X. Zhuang, S. Li, A. Young, T. Mansi, & A. Suinesiaputra (Eds.), *Statistical Atlases and Computational Models of the Heart. Multi-Sequence CMR Segmentation, CRT-EPiggy and LV Full Quantification Challenges* (pp. 364–372). Springer International Publishing. https://doi.org/10.1007/978-3-030-39074-7_38

Colli Franzone, P., Guerri, L., & Rovida, S. (1990). Wavefront propagation in an activation model of the anisotropic cardiac tissue: Asymptotic analysis and numerical simulations. *Journal of Mathematical Biology*, *28*(2), 121–176. https://doi.org/10.1007/BF00163143

Corral-Acero, J., Margara, F., Marciniak, M., Rodero, C., Loncaric, F., Feng, Y., Gilbert, A., Fernandes, J. F., Bukhari, H. A., Wajdan, A., Martinez, M. V., Santos, M. S., Shamohammdi, M., Luo, H., Westphal, P., Leeson, P., DiAchille, P., Gurev, V., Mayr, M., … Lamata, P. (2020). The 'Digital Twin' to enable the vision of precision cardiology. *European Heart Journal*, ehaa159. https://doi.org/10.1093/eurheartj/ehaa159

Dijkstra, E. W. (1959). A note on two problems in connexion with graphs. *Numerische Mathematik*, *1*(1), 269–271. https://doi.org/10.1007/BF01386390

Durrer, D., Van Dam R. Th., Freud G. E., Janse M. J., Meijler F. L., & Arzbaecher R. C. (1970). Total Excitation of the Isolated Human Heart. *Circulation*, *41*(6), 899–912. https://doi.org/10.1161/01.CIR.41.6.899

Dutta, S., Mincholé, A., Quinn, T. A., & Rodriguez, B. (2017). Electrophysiological properties of computational human ventricular cell action potential models under acute ischemic conditions. *Progress in Biophysics and Molecular Biology*, *129*, 40–52. https://doi.org/10.1016/j.pbiomolbio.2017.02.007

Gilks, W. R. (2005). Markov Chain Monte Carlo. In *Encyclopedia of Biostatistics*. American Cancer Society. https://doi.org/10.1002/0470011815.b2a14021

Gima, K., & Rudy, Y. (2002). Ionic Current Basis of Electrocardiographic Waveforms: A Model Study. *Circulation Research*, *90*(8), 889–896. https://doi.org/10.1161/01.RES.0000016960.61087.86

Konukoglu, E., Sermesant, M., Clatz, O., Peyrat, J.-M., Delingette, H., & Ayache, N. (2007). A Recursive Anisotropic Fast Marching Approach to Reaction Diffusion Equation: Application to Tumor Growth Modeling. In N. Karssemeijer & B. Lelieveldt (Eds.), *Information Processing in Medical Imaging* (pp. 687–699). Springer. https://doi.org/10.1007/978-3-540-73273-0_57

Lew, S., Wolters, C. H., Anwander, A., Makeig, S., & MacLeod, R. S. (2009). Improved EEG source analysis using low-resolution conductivity estimation in a four-compartment finite element head model. *Human Brain Mapping*, *30*(9), 2862–2878. https://doi.org/10.1002/hbm.20714

Mincholé, A., Zacur, E., Ariga, R., Grau, V., & Rodriguez, B. (2019). MRI-Based Computational Torso/Biventricular Multiscale Models to Investigate the Impact of Anatomical Variability on the ECG QRS Complex. *Frontiers in Physiology*, *10*. https://doi.org/10.3389/fphys.2019.01103

O'Hara, T., Virág, L., Varró, A., & Rudy, Y. (2011). Simulation of the Undiseased Human Cardiac Ventricular Action Potential: Model Formulation and Experimental Validation. *PLOS Computational Biology*, *7*(5), e1002061. https://doi.org/10.1371/journal.pcbi.1002061

Pezzuto, S., Kal'avský, P., Potse, M., Prinzen, F. W., Auricchio, A., & Krause, R. (2017). Evaluation of a Rapid Anisotropic Model for ECG Simulation. *Frontiers in Physiology*, *8*. https://doi.org/10.3389/fphys.2017.00265

Pitt-Francis, J., Pathmanathan, P., Bernabeu, M. O., Bordas, R., Cooper, J., Fletcher, A. G., Mirams, G. R., Murray, P., Osborne, J. M., Walter, A., Chapman, S. J., Garny, A., van Leeuwen, I. M. M.,

Maini, P. K., Rodríguez, B., Waters, S. L., Whiteley, J. P., Byrne, H. M., & Gavaghan, D. J. (2009). Chaste: A test-driven approach to software development for biological modelling. *Computer Physics Communications*, *180*(12), 2452–2471. https://doi.org/10.1016/j.cpc.2009.07.019

Potse, M. (2018). Scalable and Accurate ECG Simulation for Reaction-Diffusion Models of the Human Heart. *Frontiers in Physiology*, *9*. https://doi.org/10.3389/fphys.2018.00370

Potse, M., & Kuijpers, N. H. L. (2010). Simulation of fractionated electrograms at low spatial resolution in large-scale heart models. *2010 Computing in Cardiology*, 849–852.

Potyagaylo, D., Cortés, E. G., Schulze, W. H. W., & Dössel, O. (2014). Binary optimization for source localization in the inverse problem of ECG. *Medical & Biological Engineering & Computing*, *52*(9), 717–728. https://doi.org/10.1007/s11517-014-1176-4

Ramírez, J., Orini, M., Tucker, J. D., Pueyo, E., & Laguna, P. (2017). Variability of Ventricular Repolarization Dispersion Quantified by Time-Warping the Morphology of the T-Waves. *IEEE Transactions on Biomedical Engineering*, *64*(7), 1619–1630. https://doi.org/10.1109/TBME.2016.2614899

Schuler, S., Tate, J. D., Oostendorp, T. F., MacLeod, R. S., & Dössel, O. (2019). Spatial Downsampling of Surface Sources in the Forward Problem of Electrocardiography. In Y. Coudière, V. Ozenne, E. Vigmond, & N. Zemzemi (Eds.), *Functional Imaging and Modeling of the Heart* (pp. 29–36). Springer International Publishing. https://doi.org/10.1007/978-3-030-21949-9_4

Sörnmo, L., & Laguna, P. (2005). *Bioelectrical Signal Processing in Cardiac and Neurological Applications*. Elsevier. https://doi.org/10.1016/B978-0-12-437552-9.X5000-4

South, L. F., Pettitt, A. N., & Drovandi, C. C. (2019). Sequential Monte Carlo Samplers with Independent Markov Chain Monte Carlo Proposals. *Bayesian Analysis*, *14*(3), 753–776. https://doi.org/10.1214/18-BA1129

Tate, J. D., Schuler, S., Dössel, O., MacLeod, R. S., & Oostendorp, T. F. (2019). Correcting Undersampled Cardiac Sources in Equivalent Double Layer Forward Simulations. *Functional Imaging and*

Modeling of the Heart : ... International Workshop, FIMH ..., Proceedings. FIMH, *11504*, 147–155. https://doi.org/10.1007/978-3-030-21949-9_17

Wallman, M., Smith, N. P., & Rodriguez, B. (2012). A Comparative Study of Graph-Based, Eikonal, and Monodomain Simulations for the Estimation of Cardiac Activation Times. *IEEE Transactions on Biomedical Engineering*, *59*(6), 1739–1748. https://doi.org/10.1109/TBME.2012.2193398
